# Coordinating extreme-heat adaptation at mega-sporting events: early warning and cost sharing in a multi-actor differential game

**DOI:** 10.3389/fpubh.2026.1986768

**Published:** 2026-09-30

**Authors:** Jiaxin Zhang, Siqing Zhang, Junrong Lei

**Affiliations:** 1 College of Physical Education, Hunan Normal University, Changsha, China; 2 College of Physical Education, University of South China, Hengyang, China; 3 Department of Physical Education, Zhejiang Chinese Medical University, Hangzhou, China

**Keywords:** cost sharing, differential game, extreme heat, heat-health warning systems, mega-sporting events, multi-actor coordination, preparation window, public health preparedness

## Abstract

**Introduction:**

Coordinating extreme-heat adaptation at mega-sporting events is a time-dependent public health challenge because public authorities, event organizers, and participating teams control different protective measures while heat risk evolves over a finite competition horizon. Existing sport-heat research describes exposure and prevention yet provides limited guidance on how adaptation should be timed, coordinated, and financed across responsible institutions.

**Methods:**

We develop a three-actor linear-quadratic differential game with gross event heat-risk pressure and accumulated adaptive capacity as state variables. Open-loop Nash, government-led Stackelberg, and cooperative solutions are evaluated under four normalized heat-shock structures. Early-warning lead time enters through a piecewise information structure, and a budget-balanced cost-sharing rule supports decentralized implementation of the cooperative path.

**Results:**

In the baseline late-peak scenario with a lead time of three normalized units, cooperation reduces modeled social loss by 21.9% relative to Nash and 23.1% relative to government-led Stackelberg; peak residual heat-risk pressure falls by 28.0% and 28.7%, respectively. Under the baseline parameterization, social loss in the government-led regime exceeds the Nash value by 1.6%. Earlier warning lowers loss, residual pressure, and peak effort, although its marginal value declines once a usable preparation window is available. Across the tested combinations of warning lead time and organizer mitigation efficiency, cooperative social gains range from 16.7 to 24.4%. Within the model, the effective preparation-window threshold—defined as the first normalized lead time capturing 80% of the cooperative warning benefit available between *L* = 0 and *L* = 6—ranges from 2.2 to 2.6 and responds mainly to heat-shock duration. Cooperation also shifts effort toward organizer-controlled measures, while cost sharing compensates the organizer for carrying greater responsibility despite receiving the smallest uncompensated private gain.

**Discussion:**

The Nash—Stackelberg ordering is conditional on the government-first sequence, actor-specific objectives, and parameter values used in the analysis. Within the normalized model, these findings indicate that coordinated extreme-heat adaptation can benefit from aligning actor objectives and linking warning information to actionable time, institutional capability, and financing. Event-specific application requires empirical calibration.

## 1 Introduction

Extreme heat at mega-sporting events is a public-health preparedness challenge with consequences extending beyond athletic performance. Exposure to high temperatures can cause acute heat-related illness and exacerbate existing health conditions, making timely prevention an important component of health protection (1). At sporting mass gatherings, these concerns extend to athletes, officials, spectators, and event staff, with implications for on-site medical services and the wider health system (2). Protecting these populations requires institutional arrangements that connect environmental risk assessment and individual precautions with preventive action throughout event preparation and delivery. Recent initiatives illustrate how heat protection is becoming embedded in this process. Heat protection is increasingly reflected in both event regulations and public-health planning. Experimental studies in male football players indicate that planned breaks incorporating hydration and active cooling can attenuate thermal strain in hot conditions, supporting their inclusion in event heat-management protocols (3, 4). In December 2025, FIFA announced mandatory 3-min hydration breaks approximately midway through each half of every match at the 2026 FIFA World Cup, irrespective of weather conditions, embedding scheduled opportunities for rehydration into competition rules (5). At the broader level of mass-gathering preparedness, the World Health Organization's Beat the Heat initiative has incorporated early warning, event-specific risk assessment, escalation protocols, and mitigation measures into a heat-health action framework (6). Implementing these measures requires coordination between competition management, medical preparedness, and the institutions responsible for translating heat warnings into protective action.

Implementing these measures involves institutions with different responsibilities and intervention capabilities. Public-health authorities coordinate warning and medical readiness; event organizers manage venue-level protection and operational adjustments within competition regulations; and participating teams oversee athlete preparation and individual protective practices. Research has provided an increasingly detailed evidence base for these activities. Mullan et al. (7) examined hourly wet-bulb globe temperature across the 16 host locations of the 2026 FIFA World Cup, demonstrating the importance of match timing for heat exposure. Lindner-Cendrowska et al. (8) assessed player heat stress using an activity-adjusted Universal Thermal Climate Index across the same tournament geography. These studies identify spatial and temporal differences relevant to event planning, while the IOC consensus connects environmental assessment with competition arrangements, medical management, and athlete preparation (9). This evidence helps establish when protection is needed and which interventions are available. A further question concerns how the responsible institutions should allocate their efforts when they control different measures and bear different costs. Coordinating these decisions over a limited preparation and competition period is central to translating heat-health guidance into practice.

The timing of coordination matters because protective actions operate through different pathways and on different time scales. Adjustments to scheduling, competition breaks, and access to cooling can modify exposure during an event, whereas heat acclimation requires advance preparation and produces adaptations that persist beyond an individual training session (9, 10). Organizational readiness similarly requires resources to be committed before they are needed. Early warning can extend the opportunity for preparation, although its value depends on the actions that follow. A systematic review of heat warning systems found that warning availability did not consistently translate into behavioral change (11). For event governance, this finding motivates closer examination of how warning lead time interacts with institutional incentives to shape the timing and allocation of preventive effort.

Related emergency-coordination differential games provide a closer methodological benchmark. Yang et al. (12) examine disaster response by a government and social organizations under autonomous, government-led, and collaborative regimes and derive government cost sharing under infrastructure damage and asymmetric resource dependence. Li et al. (13) model the interaction between government and manufacturers in regular and emergency medical production and examine purchase commitments as an incentive for emergency co-production. These studies demonstrate that alternative decision sequences and financial incentives have already been incorporated into differential games of emergency management. Their settings, however, concern two-party disaster response or emergency supply production; they do not address three institutions controlling distinct heat-protection channels across the preparation and delivery of a finite sporting event.

In the event setting considered here, each actor bears its own intervention cost, while its actions alter a common risk trajectory and thereby affect the losses of the others. This creates a possible gap between individually preferred effort and joint efficiency. Government leadership changes the decision sequence but does not replace actor-specific objectives; cooperation internalizes cross-actor effects but may assign greater effort to an actor whose private benefit is modest. The unresolved issue is therefore how governance structure, warning lead time, and financing interact when both current risk pressure and accumulated preparedness evolve over time. The study examines three questions: how do decentralized, government-led, and cooperative decisions differ in the amount, timing, and composition of adaptation effort and in residual heat risk? How does warning lead time alter these outcomes under alternative heat-shock structures? Under what conditions can cost sharing support cooperation when responsibilities and private gains are unevenly distributed?

To address these questions, this study develops a finite-horizon, three-actor linear-quadratic differential game involving the government, the event organizer, and participating teams. The study extends established differential-game approaches by placing three institutional actors, warning timing, adaptive-capacity accumulation, and a budget-balanced transfer rule within the same finite-horizon model of mega-event heat-health preparedness. First, the model represents immediate risk mitigation and accumulated adaptive capacity within a common state system, allowing actor-controlled measures to provide both current and persistent protection. Second, a piecewise information structure holds the physical heat shock constant while varying the time at which event-specific information becomes available for re-optimization; this isolates actionable preparation time and supports a comparative preparation-window threshold. Third, a budget-balanced transfer rule separates marginal incentive alignment from the distribution of cooperative surplus, addressing the responsibility-benefit mismatch that can arise when cooperation relies more heavily on one actor's control. Evaluating these elements together links decision regime, warning timing, and implementation financing along the same heat-risk trajectory. The analysis therefore complements exposure assessment and intervention guidance by examining the institutional conditions under which warnings can be converted into coordinated protection. It remains conditional on normalized scenarios and does not estimate temperature, morbidity, mortality, or monetary loss; event-specific application requires empirical calibration. Section 2 reviews the relevant literature and analytical framing, Section 3 presents the model and numerical design, Section 4 reports the results, and Sections 5 and 6 discuss the implications and conclusions.

## 2 Literature and analytical framing

### 2.1 Extreme heat as an event-system risk

Heat-related health risk in sport arises from the interaction of environmental heat load, metabolic heat production, and individual thermoregulatory capacity. Activity intensity, acclimatization, age, and underlying health conditions influence the physiological consequences of exposure (1). Environmental indices provide a practical basis for assessing this exposure. Wet-bulb globe temperature (WBGT) captures the combined influence of temperature, humidity, radiant heat, and air movement, although the interpretation of its thresholds depends on the activity, clothing, and population concerned (14, 15). Activity-adjusted applications of the Universal Thermal Climate Index (UTCI) likewise show how sport-specific assumptions affect estimates of thermal stress (8). The literature thus links environmental assessment to the characteristics of the exposed population. For public-health planning, this connection is important because a common thermal environment can generate different protective needs among those attending, competing in, and working at an event.

Mass-gathering medicine extends this population perspective by examining how health needs interact with the facilities and services available at an event and in its host setting (16). In sporting events, heat exposure affects athletes, officials, spectators, volunteers, and workers, bringing individual precautions into contact with event-wide provision of water, cooling, and medical care (2). These connections are visible in medical records from the 2023 Ryder Cup, where heat-induced exhaustion was among the leading reasons for assessment among visitors and staff. The event's care arrangements linked mobile response teams and on-site facilities with regional emergency services, illustrating how health protection extended across the venue and its supporting healthcare network (17). This evidence places heat exposure within an operational setting in which access to protection and timely care helps shape its consequences. Medical-service demand is one pathway through which individual heat-related illness acquires wider significance for event delivery and local public-health resources.

These relationships underpin the use of an event-system perspective in this study. Heat constitutes a shared health-protection problem because exposed populations depend on connected protective infrastructure and care services, while the conditions affecting their safety are shaped across several institutional domains. The IOC consensus reflects this perspective by connecting environmental assessment and event arrangements with athlete preparation and medical management (9). Read alongside the mass-gathering literature, it supports an understanding of event risk that encompasses the relationship between exposure, susceptibility, and the provision of protection. The present model draws on this understanding to represent gross event heat-risk pressure as an aggregate, normalized state generated by an external heat shock. Its interpretation is confined to modeled event-level risk dynamics: WBGT, UTCI, population-specific morbidity, and medical-service utilization are not separately estimated. The broader health and service-system evidence establish the public-health context for examining institutional responses to this shared risk.

### 2.2 Dynamic adaptation and information timing

Heat-health warning systems create an opportunity to organize preventive action before hazardous conditions generate avoidable harm. Their public-health value depends on the connection between forecast information, operational readiness, and the measures available to those receiving the warning. A systematic review of heat-health action plans found substantial variation in the implementation of surveillance, monitoring, and evaluation, together with weaknesses in their integration into wider policy arrangements (18). In sport, forecasting research has similarly emphasized the practical use of anticipated thermal conditions in planning health protection during competition (19). These contributions connect the availability of heat information with the institutional capacity to act on it. For a time-limited sporting event, an important question is how much preparation remains possible when event-specific information becomes available.

The preparation required varies across protective measures. Competition adjustments and active cooling can modify exposure or physiological strain during an event, whereas heat acclimation requires repeated exposure in advance and produces adaptations that persist into subsequent activity (20). Medical readiness also depends on prior arrangements, including staff training, equipment provision, and established response procedures (9). Dynamic adaptation research provides a complementary way of examining these temporal relationships by considering how evolving conditions and new information guide intervention over time (21). Applied to event health protection, this perspective directs attention to the time available for establishing protection and the duration of its effects. The analytical framework therefore distinguishes effort that reduces current risk pressure from effort that builds adaptive capacity. Capacity-building contributes to a stock that continues to attenuate residual risk as it depreciates. A protective measure may operate through both channels, allowing the framework to accommodate interventions whose immediate effects coexist with benefits from earlier preparation.

The model examines information timing through a piecewise information structure in which actors re-optimize their remaining effort paths when event-specific information arrives. Comparisons across warning lead times hold the external heat-shock path fixed and change the re-optimization point. This design isolates the role of preparation time within the specified information structure, without attributing warning benefits to a reduction in the underlying hazard. Earlier information can change the balance between immediate mitigation and capacity-building, with the resulting benefit depending on intervention efficiency, capacity persistence, and the time remaining before exposure intensifies. The preparation-window threshold developed later summarizes this relationship as the minimum lead time required to capture a specified share of the attainable warning benefit under cooperation. It is interpreted as a planning quantity expressed in normalized time units. Its application to a particular tournament would require calibration to the event schedule and the implementation times of relevant protective measures.

### 2.3 Multi-actor coordination and decision sequence

The shared-risk setting described above brings public-health protection into decisions made under different institutional mandates. Public authorities, event organizers, and participating teams can all contribute to prevention, while bearing different costs and attaching different values to the consequences of residual risk. Research on urban heat action planning shows how institutional capacity and financing arrangements influence the implementation of protective measures, even where formal plans already identify heat as a public-health priority (22). For mega-sporting events, this raises a related question about how responsibility for prevention translates into effort when protective benefits extend across organizational boundaries. The present framework examines that question through actor-specific objectives and a common evolving risk state.

Recent evolutionary-game research provides a complementary account of why cooperative outcomes depend on the rules governing interaction. Wei et al. (23) embed collective reputation in a public-goods game and show that group-assessment criteria alter reputation dynamics, with an intermediate criterion offering the most favorable conditions for sustained cooperation. Wang et al. (24) combine analytical results with spatial simulations of a biased donation game and find that strategy-specific bias and population structure can change the long-run composition of cooperative behavior. These studies examine strategy evolution in populations. The present setting concerns a fixed set of institutions that choose protective controls over a finite event horizon. The contexts differ, but both findings underline the sensitivity of cooperation to valuation rules and interaction structure. In this study, those features enter through actor-specific loss functions, simultaneous and sequential decision regimes, and a shared heat-risk trajectory.

Differential games provide a formal basis for studying decisions that jointly affect a dynamic system under simultaneous or hierarchical decision arrangements (25). In the Nash regime, each actor chooses effort according to its own intervention cost and valuation of residual heat risk. The resulting state trajectory also affects the losses of the other actors, creating benefits that are only partly reflected in the initiating actor's objective. This dynamic externality can influence both the amount and timing of protection. Its importance depends on the effectiveness of each control, the persistence of accumulated capacity, and the remaining event horizon. Actor-specific objectives thus represent a potential source of coordination loss even when all participants have an interest in limiting heat-related harm.

Government leadership changes how these decisions are sequenced. In the Stackelberg regime, the government commits first and anticipates the responses of organizers and teams, while evaluating the resulting trajectory through its own objective. Leadership can influence preventive effort and the use of available capacity, but its effect on aggregate loss remains contingent on the model parameters and the differences among actor-specific objectives. The cooperative benchmark evaluates all three losses jointly and allocates immediate mitigation and capacity-building according to their system-wide contribution relative to effort cost. This comparison allows the analysis to assess how strategic anticipation and joint valuation of protective benefits shape the response to the same heat shock. Cooperation may also shift responsibilities toward an actor whose intervention is particularly effective, creating a distribution of effort that differs from the distribution of private gains.

Such differences make financing part of the coordination problem. Reviews of intersectoral health financing identify co-financing as a means of supporting interventions whose costs and benefits span separate budgets, while also documenting institutional barriers and limited rigorous evidence of effectiveness (26). Research on climate and health financing further emphasizes the potential for arrangements that support prevention and adaptation before adverse consequences generate expenditure elsewhere in the system (27). These findings provide an institutional rationale for examining cost sharing in event heat-health preparedness. The present model defines cost sharing through budget-balanced transfers among the three actors and distinguishes marginal incentive alignment from the redistribution of cooperative surplus. Incentive alignment concerns the effort choices needed to support the cooperative allocation; surplus redistribution concerns how the resulting gains are shared. This distinction allows the analysis to examine compensation when an actor assumes additional cooperative effort but receives a comparatively small uncompensated private gain. The mechanism's effectiveness is assessed within the model, with its practical implementation remaining dependent on the institutional arrangements of the event.

## 3 Model formulation

### 3.1 Actors, horizon, and state variables

Consider a mega-sporting event over a finite normalized planning horizon (*t* ∈ [0, *T*]). Three strategic actors represent the principal functions involved in event heat-health protection: the host government or public-health authority, indexed by *g*; the event organizer or sport governing body, indexed by *o*; and the participating team or athlete-support system, indexed by *a*. This functional grouping draws on sport-heat guidance and mass-gathering research linking health protection to event planning, environmental mitigation, medical preparedness, and participant preparation (2, 9). Each actor represents responsibilities that may be distributed across several institutions in practice.

Actor *i* chooses a nonnegative adaptation intensity *u*_*i*_(*t*)≥0 in continuous time. Government effort encompasses warning communication, public-health preparedness, and medical response readiness. Organizer effort includes scheduling, venue-level mitigation, hydration and cooling arrangements, and rule-based operational changes. Team effort covers heat acclimation, hydration preparation, recovery, monitoring, and athlete-support activities. Each control summarizes the intensity of an actor's protective activities, with its effects represented through actor-specific coefficients. In the early-warning extension, the arrival time of event-specific information is specified separately; government effort concerns preparedness and the use of that information.

Two state variables summarize the evolving conditions for heat-health protection. Gross event heat-risk pressure, *E*(*t*), captures pressure before accounting for protection supplied by accumulated adaptive capacity, *A*(*t*). Its evolution already incorporates the effects of immediate mitigation. Residual heat-risk pressure is defined as


$$\begin{array}{l} R\left(t\right)=E\left(t\right)-\kappa A\left(t\right),\;\kappa >0. \end{array}$$ (1)

In Equation 1, the coefficient κ scales the protective contribution of adaptive capacity. Both states and the resulting residual-risk measure are dimensionless constructs. Their normalization supports comparisons of adaptation strategies under common assumptions, without assigning them units of temperature, WBGT, illness incidence, or monetary loss. The analysis therefore concerns the organization of heat-health protection under stylized conditions, with no empirical calibration to a particular tournament or venue.

The state dynamics are


$$\begin{array}{l} Ė\left(t\right)=-\delta E\left(t\right)+\beta H\left(t\right)-\gamma_{g}u_{g}\left(t\right)-\gamma_{o}u_{o}\left(t\right)-\gamma_{a}u_{a}\left(t\right), \end{array}$$ (2)

$$\begin{array}{l} Ȧ\left(t\right)=-\rho A\left(t\right)+\eta_{g}u_{g}\left(t\right)+\eta_{o}u_{o}\left(t\right)+\eta_{a}u_{a}\left(t\right). \end{array}$$ (3)

Here, *H*(*t*) denotes the exogenous normalized heat shock, β converts this forcing into gross risk pressure, and δ governs the natural dissipation of that pressure. The coefficient γ_*i*_ measures the immediate mitigation efficiency of actor *i*, while η_*i*_ measures its contribution to accumulated adaptive capacity. Capacity depreciates at rate ρ. Equation 2 represents actions that reduce exposure-related or operational pressure while leaving the external heat forcing unchanged. Equation 3 represents preparation whose protective contribution remains available after the effort has been supplied. A single intervention can contribute through both channels.

The stock formulation accommodates the persistence of preparation, an important feature of measures such as heat acclimation. Evidence on the retention and subsequent decay of acclimation benefits provides a physiological example of this temporal structure (10). Aggregating physiological preparation and organizational readiness into one state with a constant depreciation rate is a simplifying modeling assumption. The coefficients describe the effectiveness and persistence assigned to these channels within the normalized scenarios.

For compact notation, let *x*(*t*) = [*E*(*t*), *A*(*t*)]^⊤^, *C* = [1, −κ], $B_{i}={\left[-\gamma_{i},\eta_{i}\right]}^{⊤}$, *d* = [β, 0]^⊤^, and


$$\begin{array}{l} F=\left[\begin{array}{cc} -\delta & 0\\ 0 & -\rho \end{array}\right]. \end{array}$$

The system can then be written as


$$\begin{array}{l} ẋ\left(t\right)=Fx\left(t\right)+B_{g}u_{g}\left(t\right)+B_{o}u_{o}\left(t\right)+B_{a}u_{a}\left(t\right)+dH\left(t\right),\\ R\left(t\right)=Cx\left(t\right). \end{array}$$ (4)

Equation 4 expresses the state dynamics and residual-risk measure in compact matrix form.

### 3.2 Heat-shock structure

The external forcing H(t) is specified through normalized profiles that vary in amplitude, timing, and temporal width. These features determine when protective measures are needed and how long preparedness must be maintained. The operational importance of temporal variation is illustrated by hourly heat-risk assessments across the 2026 FIFA World Cup host locations, which identify substantial within-day differences in environmental heat stress (7). In the present analysis, stylized profiles provide controlled conditions for comparing governance arrangements.

A single-peak heat shock is represented by


$$\begin{array}{l} H\left(t\right)=h_{0}+h_{1}\exp\left[-\frac{1}{2}{\left(\frac{t-t_{p}}{\sigma }\right)}^{2}\right]. \end{array}$$ (5)

where *h*_0_ is the baseline forcing, *h*_1_ is the peak amplitude above that baseline, and *t*_*p*_ determines peak timing. The positive parameter σ controls the temporal width of the elevated forcing and thereby its persistence within the planning horizon.

A double-peak scenario combines two Gaussian components:


$$\begin{array}{l} H\left(t\right)=h_{0}+\overset{2}{\underset{m=1}{\sum }}h_{m}\exp\left[-\frac{1}{2}{\left(\frac{t-t_{p,m}}{\sigma_{m}}\right)}^{2}\right]. \end{array}$$ (6)

For component m, *h*_*m*_ denotes its amplitude, *t*_*p, m*_ its peak timing, and σ_*m*_ its temporal width. The combined profile permits successive episodes of elevated forcing, allowing the analysis to examine the contribution of protective capacity retained between peaks.

Equations 7, 6 generate deterministic scenarios representing a routine hot period, a persistent heatwave, a late concentrated peak, and a double-peak sequence. Within each scenario, the same external forcing is applied across governance regimes. This makes differences in modeled outcomes attributable to adaptation decisions under the specified institutional arrangements. The Gaussian functions serve as scenario-generating devices; they carry no probability interpretation and are not fitted to observed meteorological series. Their purpose is to examine how the timing and persistence of heat pressure affect the allocation of protective effort.

### 3.3 Actor-specific objectives

Each actor evaluates the residual heat-risk pressure associated with the shared event state and the resource burden of its own adaptation effort. Differences in institutional responsibilities are represented through actor-specific risk weights and effort-cost coefficients. The objective for actor *i* is


$$\begin{array}{l} J_{i}=\frac{1}{2}\int_{0}^{T}\left[q_{i}R{\left(t\right)}^{2}+c_{i}u_{i}{\left(t\right)}^{2}\right]dt+\frac{1}{2}\phi_{i}R{\left(T\right)}^{2},\;i\in \left\{g,o,a\right\}. \end{array}$$ (7)

The parameter (*q*_*i*_ > 0) weights residual heat-risk pressure throughout the planning horizon, (*c*_*i*_ > 0) is the quadratic effort-cost coefficient, and (ϕ_*i*_ > 0) weights residual pressure at the terminal time. Over the positive-risk range, the quadratic specification assigns increasing marginal penalties to residual pressure and to more intensive effort. It provides a tractable representation of the trade-off between heat protection and the resources required to deliver it. Its curvature is a modeling assumption, with no empirical calibration to a heat–morbidity relationship.

The risk weights encode the institutional sensitivity assigned to the common state. For the public authority, this concerns event-wide health protection and preparedness; for the organizer, the safety-related consequences of event operations; and for the team, the protection of participating athletes. Differences in *c*_*i*_ allow the same normalized effort intensity to impose different resource burdens across actors. The terminal term gives weight to risk that remains unresolved at the end of the decision window. It does not introduce a separate model of post-event illness or healthcare utilization.

Define ($Q_{i}=q_{i}C^{⊤}C$) and ($\phi_{i}=\phi_{i}C^{⊤}C$). Equation 7 then has the standard linear-quadratic form, with *Q*_*i*_ and ϕ_*i*_ weighting the state vector through its contribution to residual heat-risk pressure.

The model's social-loss criterion is the sum of the three actor-specific objectives:


$$\begin{array}{l} J_{\Sigma }=J_{g}+J_{o}+J_{a}. \end{array}$$ (8)

Equation 8 combines weighted residual-risk pressure and effort costs in normalized units. It provides a common criterion for comparing decentralized decisions, government leadership, and cooperation. Its numerical value should be interpreted as modeled aggregate loss, without a direct conversion to monetary expenditure or cases of heat-related illness.

The public authority and organizer are interpreted as bearing responsibilities toward spectators, workers, volunteers, and other attendees, consistent with the broader populations considered in mass-gathering heat-health research (2). These groups have no separate strategic controls or population-specific health equations, so their protection enters only through the institutional interpretation of the risk weights. The model consequently supports comparisons of institutional effort allocation while leaving differences in vulnerability and health outcomes across population groups unresolved. This aggregation is revisited in Section 6.6.

### 3.4 Open-loop Nash equilibrium

Under open-loop Nash behavior, each actor selects an effort path *u*_*i*_(*t*) that minimizes *J*_*i*_, taking the other actors' planned paths as given. Decisions are conditional on the initial state and the heat-shock information available at the beginning of the decision stage, with controls specified as functions of time (25). In the early-warning extension, the remaining-horizon problem is solved again when event-specific information arrives; the open-loop characterization applies within each information stage.

The equilibrium conditions follow the Hamiltonian approach to finite-horizon optimal control (28). Let ($\lambda_{i}\left(t\right)\in {\mathbb{R}}^{2}$) denote actor *i*'s costate vector. Its components measure the marginal contribution of gross risk pressure and adaptive capacity to that actor's remaining objective. The Hamiltonian is


$$\begin{array}{l} H_{i}=\frac{1}{2}x^{⊤}Q_{i}x+\frac{1}{2}c_{i}u_{i}^{2}+\lambda_{i}^{⊤}\left(Fx+B_{g}u_{g}+B_{o}u_{o}+B_{a}u_{a}+dH\right). \end{array}$$ (9)

Equation 9 defines the actor-specific Hamiltonian used to derive the first-order condition for effort.

For an interior optimum with respect to effort, the first-order condition gives


$$\begin{array}{l} u_{i}^{N}\left(t\right)=-\frac{B_{i}^{⊤}\lambda_{i}\left(t\right)}{c_{i}}. \end{array}$$ (10)

The superscript *N* denotes the Nash regime. Equation 10 characterizes the interior control; its application over the full horizon requires the resulting effort paths to satisfy the nonnegativity restriction. Binding control constraints are outside this interior characterization.

The corresponding costate dynamics and terminal conditions are


$$\begin{array}{l} {\dot{\lambda }}_{i}\left(t\right)=-Q_{i}x\left(t\right)-F^{⊤}\lambda_{i}\left(t\right),\;\lambda_{i}\left(T\right)=\phi_{i}x\left(T\right). \end{array}$$ (11)

The terminal condition follows from the residual-risk penalty at *T*, while the costate dynamics account for the contribution of the evolving state to subsequent losses. Together with Equation 10, these conditions connect current adaptation effort to its consequences throughout the remaining decision horizon.

Let ($M_{i}=B_{i}B_{i}^{⊤}/c_{i}$). Combining the state equation with the three costate systems yields the two-point boundary-value problem in Equation 12


$$\begin{array}{l} \frac{d}{dt}\left[\begin{array}{c} x\\ \lambda_{g}\\ \lambda_{o}\\ \lambda_{a} \end{array}\right]=\left[\begin{array}{cccc} F & -M_{g} & -M_{o} & -M_{a}\\ -Q_{g} & -F^{⊤} & 0 & 0\\ -Q_{o} & 0 & -F^{⊤} & 0\\ -Q_{a} & 0 & 0 & -F^{⊤} \end{array}\right]\left[\begin{array}{c} x\\ \lambda_{g}\\ \lambda_{o}\\ \lambda_{a} \end{array}\right]+\left[\begin{array}{c} dH\\ 0\\ 0\\ 0 \end{array}\right]. \end{array}$$ (12)

The initial state *x*(0) = *x*_0_ is fixed, and the terminal costates satisfy Equation 11. The two initial state conditions and six scalar terminal conditions specify the boundaries of this eight-dimensional state–costate system. Subject to the admissibility of the resulting controls, the solution provides the decentralized benchmark without a leader or transfer mechanism.

In this benchmark, each actor balances its effort cost against the reduction in its own weighted heat-risk loss. Protective benefits entering the objectives of other actors receive no additional weight in its first-order condition. An effective adaptation channel can consequently be weakly incentivized when a substantial share of its benefits accrues beyond the institution supplying the effort. This connects the Nash formulation to the public-health problem of coordinating protective activities across institutions with shared exposure but separate responsibilities and resource commitments.

### 3.5 Government-led open-loop Stackelberg equilibrium

The government-led regime represents a public authority that commits an event-wide preparedness path before the organizer and participating teams finalize their responses. In the context of heat management at mega-sporting events, a host government or public-health authority may establish safety requirements, commit public-health and emergency resources, and activate cross-agency coordination before venue-level and team-level measures are finalized. Leadership is defined through this sequence of institutional commitment. Information remains symmetric: the event-specific heat profile becomes available to all three actors at the same warning time *t*_*w*_. The baseline continues to assign the organizer the highest direct-mitigation efficiency, consistent with its control over scheduling and venue measures. Government leadership therefore describes the authority's capacity to initiate an event-wide preparedness framework, while intervention effectiveness and operational control remain actor specific. Conditional on the government's commitment, the organizer and team choose mutually consistent open-loop best responses according to their own objectives. This structure is a specific application of the multi-player Stackelberg formulation (29).

Let λ_*o*_ and λ_*a*_ denote the organizers and team's costate vectors. The government minimizes its own objective subject to the state dynamics and the followers' optimality conditions. Its augmented optimal-control problem introduces the two-dimensional adjoint vectors *p*_*x*_, *p*_*o*_, and *p*_*a*_, associated respectively with the shared state and the two follower costate systems. Using the definitions of *Q*_*i*_, ϕ_*i*_, and *M*_*i*_ established above, the interior necessary conditions are given in Equations 13–16


$$\begin{array}{l} ẋ=Fx-M_{o}\lambda_{o}-M_{a}\lambda_{a}-M_{g}p_{x}+dH, \end{array}$$ (13)

$$\begin{array}{l} {\dot{\lambda }}_{o}=-Q_{o}x-F^{⊤}\lambda_{o},\;{\dot{\lambda }}_{a}=-Q_{a}x-F^{⊤}\lambda_{a}, \end{array}$$ (14)

$$\begin{array}{l} {ṗ}_{x}=-Q_{g}x-F^{⊤}p_{x}+Q_{o}p_{o}+Q_{a}p_{a}, \end{array}$$ (15)

$$\begin{array}{l} {ṗ}_{o}=M_{o}p_{x}+Fp_{o},\;{ṗ}_{a}=M_{a}p_{x}+Fp_{a}. \end{array}$$ (16)

The initial state remains fixed at *x*(0) = *x*_0_. The remaining boundary conditions are given in Equations 17, 18


$$\begin{array}{c} \lambda_{o}\left(T\right)=\phi_{o}x\left(T\right),\;\lambda_{a}\left(T\right)=\phi_{a}x\left(T\right),\\ p_{o}\left(0\right)=p_{a}\left(0\right)=0, \end{array}$$ (17)

$$\begin{array}{l} p_{x}\left(T\right)=\phi_{g}x\left(T\right)-\phi_{o}p_{o}\left(T\right)-\phi_{a}p_{a}\left(T\right). \end{array}$$ (18)

The follower terminal conditions retain their actor-specific valuation of residual risk. The zero initial conditions for *p*_*o*_ and *p*_*a*_ arise because the initial follower costates are free in the augmented problem. Equation 18 accounts for the dependence of the follower terminal costates on the terminal state.

The corresponding equilibrium controls are given in Equation 19


$$\begin{array}{l} u_{g}^{S}=-\frac{B_{g}^{⊤}p_{x}}{c_{g}},\;u_{o}^{S}=-\frac{B_{o}^{⊤}\lambda_{o}}{c_{o}},\;u_{a}^{S}=-\frac{B_{a}^{⊤}\lambda_{a}}{c_{a}}. \end{array}$$ (19)

The superscript *S* denotes the Stackelberg regime. As in the Nash formulation, these expressions characterize interior controls and apply over the full horizon when the resulting effort paths satisfy the nonnegativity restriction.

The government's commitment influences adaptation through its own protective effort and the responses anticipated from the other institutions. Its objective remains *J*_*g*_, so the resulting allocation reflects the public authority's assigned risk valuation alongside the followers' separate priorities. The sign of ($J_{\Sigma }^{S}-J_{\Sigma }^{N}$) is therefore determined by the actor-specific objectives, intervention efficiencies, effort costs, state dynamics, and resulting equilibrium paths. Because these characteristics differ across the three actors, the comparison applies specifically to the government-first hierarchy examined here. Assigning leadership to the organizer or team would define a separate equilibrium problem and may produce a different allocation.

### 3.6 Cooperative social optimum

Cooperation represents joint planning of event heat-health protection across the three actors. Research on intersectoral collaboration for population health emphasizes the importance of shared purposes, adequate resources, and governance arrangements that sustain coordinated action (30). The model formalizes this alignment through joint minimization of *J*_Σ_, incorporating all three actor-specific losses into the allocation of adaptation effort.

The aggregate running and terminal state-weight matrices are defined in Equation 20


$$\begin{array}{l} Q_{C}=Q_{g}+Q_{o}+Q_{a},\;\phi_{C}=\phi_{g}+\phi_{o}+\phi_{a}. \end{array}$$ (20)

Let λ_*C*_ denote the two-dimensional cooperative costate vector. The interior optimal controls are


$$\begin{array}{l} u_{i}^{C}\left(t\right)=-\frac{B_{i}^{⊤}\lambda_{C}\left(t\right)}{c_{i}},\;i\in \left\{g,o,a\right\}. \end{array}$$ (21)

The superscript *C* denotes cooperation. Each actor's effort now responds to a common valuation of the shared state, while its mitigation efficiency, capacity-building contribution, and effort-cost coefficient remain actor-specific.

The cooperative state–costate system is


$$\begin{array}{l} ẋ=Fx-\left(M_{g}+M_{o}+M_{a}\right)\lambda_{C}+dH, \end{array}$$ (22)

$$\begin{array}{l} {\dot{\lambda }}_{C}=-Q_{C}x-F^{⊤}\lambda_{C},\;\lambda_{C}\left(T\right)=\phi_{C}x\left(T\right). \end{array}$$ (23)

Together with *x*(0) = *x*_0_, and the admissible controls in Equations 21–23 characterize the cooperative path.

For a fixed initial state, specified heat-shock path, and common information set, joint minimization over the same admissible controls provides the aggregate-loss benchmark. Its advantage may arise through changes in total effort, the timing of preparation, or the distribution of responsibilities across institutions. In public-health terms, the common costate incorporates the modeled consequences of protective action across all three objectives, allowing immediate mitigation and accumulated preparedness to be evaluated together.

This benchmark remains conditional on the normalized risk weights and intervention coefficients. The distribution of responsibilities generated by joint optimization can differ from the distribution of actor-specific gains. Section 3.8 examines how a contractual sharing rule can reconcile these differences. It should therefore be interpreted as an idealized system benchmark whose decentralized implementation requires a shared objective and enforceable transfers or comparable coordination arrangements, rather than as an equilibrium advantage arising independently of the model design.

### 3.7 Early-warning information structure

Heat-health warning systems connect environmental information with protective decisions, and their design also depends on local epidemiological evidence and population vulnerability (31). The present model focuses on the timing component of this process: when event-specific information becomes available for revising preparedness and mitigation plans.

Let *t*_*h*_ denote the timing of the heat peak used as the warning reference and *t*_*w*_ the arrival time of event-specific information. Warning lead time is


$$\begin{array}{l} L=t_{h}-t_{w},\;L\geq 0. \end{array}$$ (24)

All time variables in Equations 24, 25 are expressed on the normalized event horizon. Warning lead time (L) therefore identifies a relative position within (*T* = 10). Conversion into hours or days requires a separate temporal mapping based on the schedule and planning period of the event under consideration.

For a single-peak scenario, *t*_*h*_ corresponds to the peak timing specified in Section 3.2. For a multi-peak scenario, it denotes the selected reference peak, which is held fixed within each lead-time comparison. The piecewise implementation below presumes that warning arrival occurs within the modeled horizon. A zero-lead time means that information arrives at the reference peak, with subsequent decisions still available for revision.

Before *t*_*w*_, actors follow effort paths optimized under a baseline forecast containing only *h*_0_. These plans are implemented without updating the event-specific shock information during the pre-warning stage, while the realized model state evolves under the actual *H*(*t*). At *t*_*w*_, the event-specific shock profile is revealed and the current state *x*(*t*_*w*_) is taken as known. Each governance regime then re-solves its remaining-horizon problem over [*t*_*w*_, *T*], using that realized state as its initial condition. The implemented control is


$$\begin{array}{l} u_{i}\left(t;L\right)=\{\begin{array}{c} u_{i}^{base}\left(t\right),\&0\leq t<t_{w},\\ u_{i}^{shock}\left(t|x\left(t_{w}\right)\right),\&t_{w}\leq t\leq T. \end{array} \end{array}$$ (25)

Governance-regime superscripts are suppressed in Equation 25; both segments are calculated under the same regime. The state is carried continuously across the information update, while effort can change when the remaining-horizon plan is revised.

This specification is a deterministic information-switching experiment. It assumes that all three actors receive the event-specific profile at the same time and that the revealed profile is accurate. Forecast errors, false alarms, and differences in information uptake are outside the experiment. The physical forcing *H*(*t*) is held constant across values of *L*, allowing the analysis to examine how additional preparation time is used under otherwise unchanged heat conditions.

The implemented policy consists of stage-specific open-loop solutions. For the Stackelberg regime, re-solving at *t*_*w*_ is interpreted as a renewed government commitment for the remaining horizon. This distinction matters because commitment and re-optimization can generate different outcomes in dynamic leadership games (32). The resulting comparisons measure the effect of warning timing under the specified re-optimization protocol; in the Stackelberg case, they also reflect when remaining-horizon commitments are renewed.

### 3.8 Cost-sharing coordination

Joint planning can improve aggregate heat-health protection while placing additional responsibilities on an actor whose uncompensated gain is relatively small. This creates an institutional financing problem when the benefits of prevention extend beyond the organization supplying the resources. Evidence on intersectoral health financing identifies differences in incentives, accountability, and sector-specific benefits as important considerations in designing joint funding arrangements (33). In the event setting, cost sharing concerns the allocation of modeled costs among the institutions responsible for prevention and preparedness.

To align decentralized incentives with the cooperative objective, define actor (i)'s contracted objective as


$$\begin{array}{l} {\tilde{J}}_{i}=\theta_{i}J_{\Sigma }+K_{i},\;\theta_{i}>0,\;\underset{i}{\sum }\theta_{i}=1. \end{array}$$ (26)

The parameter θ_*i*_ determines the actor's contractual weight on aggregate loss, while *K*_*i*_ is a fixed adjustment with respect to its control. Each contracted objective is thus a positive affine transformation of the same aggregate criterion. Holding the other actors' controls fixed, an actor cannot lower its contracted loss by departing unilaterally from a cooperative optimum. Equivalently, its first-order condition is proportional to the corresponding condition for joint optimization.

This incentive interpretation requires Equation 26 to apply to the loss generated by the implemented actions and resulting state path. The contract therefore presumes agreed valuation rules, sufficient observability for settlement, and enforcement of the specified allocation. The transfer evaluated at the cooperative path below records the resulting settlement; a fixed payment independent of subsequent behavior would not provide the same marginal incentives.

All benchmark losses used in the sharing rule refer to the same heat-shock scenario, initial state, and warning lead time. The Nash outcome serves as the outside option. Define


$$\begin{array}{l} \Delta =J_{\Sigma }^{N}-J_{\Sigma }^{C}. \end{array}$$ (27)

as the cooperative surplus measured by avoided aggregate loss. For comparisons with (Δ ≥ 0), let (ω_*i*_ ≥ 0) denote actor *i*'s surplus share, with ($\underset{i}{\sum }\omega_{i}=1$), and choose


$$\begin{array}{l} K_{i}=J_{i}^{N}-\theta_{i}J_{\Sigma }^{C}-\omega_{i}\Delta . \end{array}$$ (28)

The benchmark quantities in Equation 28 are held fixed when evaluating an actor's unilateral change in effort. The parameters θ_*i*_ and ω_*i*_ perform distinct roles: the former scale the common marginal objective, while the latter allocate the cooperative surplus.

At the cooperative path, contracted loss becomes


$$\begin{array}{l} {\tilde{J}}_{i}^{C}=J_{i}^{N}-\omega_{i}\Delta \leq J_{i}^{N}. \end{array}$$ (29)

Under (Δ ≥ 0) and nonnegative surplus shares, Equation 29 satisfies the participation condition relative to Nash over the contracted horizon. An actor obtains a strict improvement when its allocated surplus is positive. This is a horizon-level participation result and presumes commitment to the agreed rule during implementation.

The net transfer evaluated at the cooperative path is


$$\begin{array}{l} T_{i}={\tilde{J}}_{i}^{C}-J_{i}^{C}. \end{array}$$ (30)

Here, $J_{i}^{C}$ is the actor's loss under the cooperative effort path before contractual redistribution, and ${\tilde{J}}_{i}^{C}$ is its loss after that redistribution. A positive *T*_*i*_ denotes a net payment, while a negative value denotes compensation. These quantities are expressed in the model's common normalized loss units; an operational funding arrangement would require an additional mapping to actual institutional budgets.

Equation 26 can be interpreted as an ex-ante resource-sharing agreement among the public authority, event organizer, and participating teams. The coefficient θ_*i*_ specifies actor (i)'s contractual share of the jointly evaluated objective, thereby linking its decision to the consequences of the implemented plan for the event system. The fixed adjustment *K*_*i*_ allocates the cooperative surplus and supports participation without changing the marginal condition governing effort. At the cooperative path, *T*_*i*_ records the actor's net settlement position. A positive value corresponds to a resource contribution, whereas a negative value corresponds to compensation for assuming responsibilities whose modeled benefits extend to other institutions or participant groups. The settlement may be delivered through financial contributions, reimbursement of verified expenditures, shared procurement, or in-kind commitments of personnel, facilities, equipment, and medical capacity. A pooled fund or reimbursement arrangement provides the payment vehicle, while the contingent sharing rule links settlement to the jointly implemented plan and its agreed evaluation.

Given the normalizations of θ_*i*_ and ω_*i*_, Equations 27, 28 imply ($\underset{i}{\sum }K_{i}=0$). Together with Equations 26, 30, this yields $\underset{i}{\sum }T_{i}=0$, preserving budget balance through internal redistribution. The baseline experiment uses equal marginal and surplus shares, θ_*i*_ = ω_*i*_ = 1/3. This transparent contractual benchmark leaves protective effort determined by actor-specific effectiveness and costs, so equal shares can coexist with unequal responsibilities and different net transfers.

## 4 Numerical design

### 4.1 Normalized parameterization

The numerical analysis uses a normalized scenario experiment to examine how institutional responsibilities, intervention effectiveness, and effort costs shape heat-health protection at a mega-sporting event. Table 1 specifies the baseline parameters for comparing governance regimes. Evidence from sport medicine and public-health research informs the interpretation of the adaptation channels, while coefficient magnitudes and cross-actor rankings are specified as scenario assumptions. The parameterization has no empirical calibration to a particular tournament, meteorological series, health-outcome dataset, or financial accounting system. The sensitivity analyses reported below examine how the findings change under the specified parameter variations.

**Table 1:** Baseline normalized parameters.

Parameter	Value	Interpretation
*T*	10.00	Normalized event horizon
β	1.00	Heat-forcing conversion coefficient
δ	0.35	Gross risk-pressure dissipation rate
ρ	0.15	Adaptive-capacity depreciation rate
κ	0.60	Protective effect of adaptive capacity
γ_*g*_	0.08	Government direct mitigation efficiency
γ_*o*_	0.20	Organizer direct mitigation efficiency
γ_*a*_	0.05	Team direct mitigation efficiency
η_*g*_	0.22	Government adaptive-capacity efficiency
η_*o*_	0.16	Organizer adaptive-capacity efficiency
η_*a*_	0.26	Team adaptive-capacity efficiency
*q*_*g*_	2.00	Government running residual-risk weight
*q*_*o*_	1.40	Organizer running residual-risk weight
*q*_*a*_	2.20	Team running residual-risk weight
*c*_*g*_	7.50	Government effort-cost coefficient
*c*_*o*_	8.50	Organizer effort-cost coefficient
*c*_*a*_	6.50	Team effort-cost coefficient
ϕ_*g*_	2.20	Government terminal residual-risk weight
ϕ_*o*_	1.60	Organizer terminal residual-risk weight
ϕ_*a*_	2.40	Team terminal residual-risk weight

The baseline coefficients were selected to create functionally distinct actor profiles within a common normalized scale. Direct mitigation follows (γ_*o*_>γ_*g*_>γ_*a*_). The organizer receives the largest coefficient because scheduling, venue operations, cooling provision, and competition breaks can alter exposure across the event population (2, 9). Government action has a moderate immediate effect through public communication, emergency-service deployment, and medical readiness. Team action is directed mainly toward athletes and therefore receives the smallest event-wide direct coefficient. Capacity-building follows (η_*a*_>η_*g*_>η_*o*_). This ordering emphasizes acclimation, monitoring, and individualized preparation managed by teams, followed by public-health and medical preparedness coordinated by government (34, 35). Organizer effort retains a smaller capacity component for pre-positioned resources and operating arrangements that remain available during the event. Its principal modeled effect is immediate operational control. The resulting profiles emphasize organizer-led direct mitigation, team-led preparation, and a mixed government role. Empirical comparison of institutional effectiveness would require event-specific data.

Effort costs follow (*c*_*o*_>*c*_*g*_>*c*_*a*_). The organizer coefficient reflects the logistical expense and disruption associated with venue and schedule changes. The government coefficient represents the resource demands of coordination, warning delivery, and medical mobilization. The team coefficient reflects the narrower operational scale assigned to athlete preparation in the baseline; actual costs may vary across sports, teams, and event settings. Running and terminal risk weights follow (*q*_*a*_>*q*_*g*_>*q*_*o*_) and (ϕ_*a*_>ϕ_*g*_>ϕ_*o*_). The team and government receive the two higher, closely spaced weights to represent direct athlete protection and event-wide public-health responsibility, respectively. The organizer's lower weight gives operational burden greater relative prominence in its objective. These weights describe modeled decision priorities; ethical responsibility, population vulnerability, and institutional preferences require separate empirical assessment. Terminal weights are set slightly above the corresponding running weights so that unresolved risk at (T) remains consequential. The condition (ρ = 0.15 < δ = 0.35) allows adaptive capacity to decay more slowly than gross risk pressure, preserving part of the benefit of earlier preparation. All governance regimes use the same parameter set, enabling comparison under a common institutional profile.

The initial state is $x_{0}={\left[0.12,0.05\right]}^{⊤}$. The resulting control paths are evaluated against the admissible interval [0, 2] for each actor. All evaluated controls satisfy these bounds. Residual-risk nonnegativity is checked separately as an output-state diagnostic rather than as a solver convergence criterion; the central regime comparisons remain nonnegative, whereas some auxiliary L = 6 threshold-reference paths can cross slightly below zero in the unconstrained linear-quadratic state system. The open-loop first-order conditions are written as two-point state–costate boundary-value problems and solved in MATLAB R2024b with the collocation solver bvp4c. The full-horizon initialization uses 121 equally spaced mesh points; re-optimization subwindows use a proportional mesh with at least 25 points, and reported paths are evaluated on 501 points over the normalized horizon *T* = 10. The relative and absolute error tolerances are 10^−6^ and 10^−8^, respectively, with a maximum of 20,000 mesh nodes. The pre-warning state is propagated with ode45 at the same tolerances, and baseline controls are evaluated by shape-preserving cubic interpolation. A run is retained when the solvers terminate within these settings; post-solution checks then verify the [0, 2] control interval and the residual-risk path.

### 4.2 Heat scenarios and baseline case

Table 2 and Figure 1 present four normalized heat-shock scenarios for examining the timing and continuity of event heat-health protection. Population-level evidence of acute and lagged associations between heatwaves and mortality supports attention to how protective responses unfold over time (36). S1 provides a relatively broad, moderate peak under routine hot conditions. S2 combines stronger forcing with longer persistence, allowing sustained adaptation requirements to be examined. S3 introduces a later, narrower peak that provides a setting for evaluating advance preparation. S4 contains two successive peaks to examine whether comparisons among governance regimes remain consistent under recurrent heat pressure. These profiles represent stylized exposure conditions, with no empirical calibration to meteorological records or health outcomes.

**Table 2:** Normalized heat-shock scenarios.

Scenario	Label	*h*_0_	Peak amplitude(s)	Peak time(s)	Width(s)
S1	Routine hot conditions	0.18	0.85	6.0	1.60
S2	Persistent heatwave	0.22	1.00	6.0	2.20
S3	Late sudden peak	0.18	1.05	7.8	1.10
S4	Double-peak heatwave	0.16	0.65; 0.75	4.2; 7.4	0.90; 1.00

**Figure 1:**
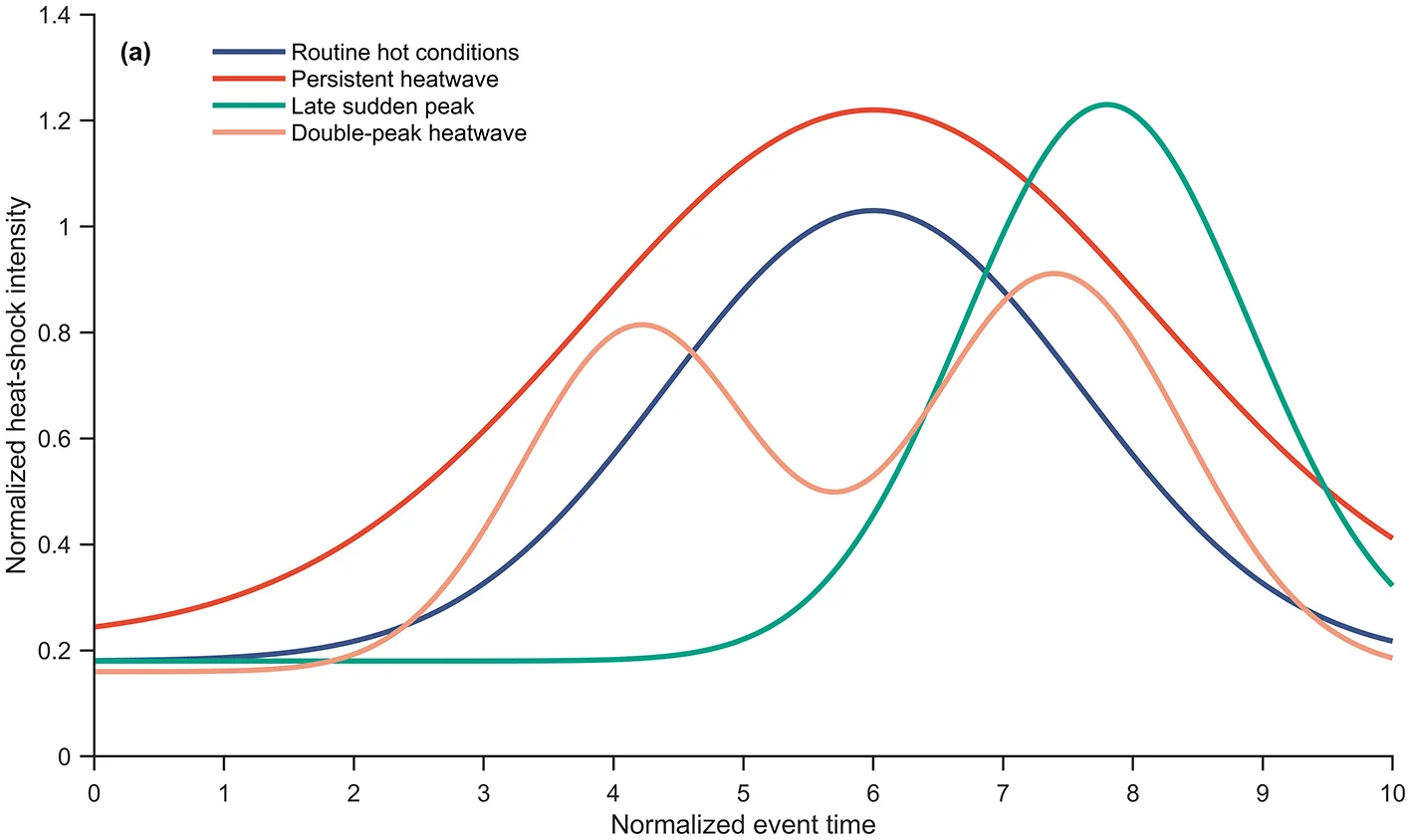
Normalized external heat-shock scenarios used in the numerical analysis. The profiles are scenario forcing functions and are not WBGT or temperature series.

The baseline uses S3 with a warning lead time of (*L* = 3), giving information arrival at (*t*_*w*_ = 4.8) before the heat peak at (*t*_*h*_ = 7.8). Within the normalized horizon (T=10), this configuration retains both a substantial pre-warning interval and a three-unit preparation window before the peak. Following Section 3.7, actors revise their remaining horizon plans at *t*_*w*_, using the revealed shock profile and the current state. This setting allows the three governance regimes to be compared in terms of how they allocate preparedness and immediate mitigation to protect event participants under the same external heat shock.

### 4.3 Outcome measures and robustness checks

The numerical analysis reports two principal system-level outcomes in normalized model units. Residual heat-risk pressure, *R*(*t*) = *E*(*t*)−κ*A*(*t*), is a dimensionless index of unresolved event-level pressure after the modeled protective contribution of accumulated adaptive capacity has been incorporated. It has no direct scale in air temperature, WBGT, UTCI, illness probability, or numbers of medical cases. Aggregate social loss, *J*_Σ_, is the sum of the three actor-specific objective values and combines weighted squared residual pressure with normalized effort costs over the planning horizon. It is not a monetary welfare measure or an estimate of economic loss. Lower levels and percentage differences are used to compare governance regimes under the same parameterization; they do not quantify corresponding changes in observed health or economic outcomes. Peak residual heat-risk pressure, R(t), and terminal residual pressure, *R*(*T*), summarize the maximum and end-of-horizon values of the modeled pressure index.

Cumulative effort by actor *i* is $U_{i}=\int_{0}^{T}u_{i}\left(t\right),dt$. When total cumulative effort is positive, the actor's responsibility share is


$$\begin{array}{c} s_{i}=\frac{U_{i}}{U_{g}+U_{o}+U_{a}}. \end{array}$$ (31)

The measure *s*_*i*_ describes the operational distribution of modeled protective effort; it does not assign legal liability or represent the distribution of health outcomes. The temporal center of adaptation effort is measured by the Adaptation Timing Index defined in Equation 32:


$$\begin{array}{c} ATI_{i}=\frac{\int_{0}^{T}tu_{i}\left(t\right)dt}{T\int_{0}^{T}u_{i}\left(t\right)dt}. \end{array}$$ (32)

For *U*_*i*_ > 0 and nonnegative effort, *ATI*_*i*_ ∈ [0, 1], with a lower value indicating that a larger share of effort is supplied earlier in the event horizon. Peak effort, u_i_(t), measures the maximum concentration of response demand placed on an actor. These effort indicators are expressed in normalized units and should not be interpreted as observed expenditure, staffing, or medical-service utilization.

Warning lead time is varied from 0 to 6 in one-unit increments. The one-way sensitivity analysis applies multipliers of 0.70, 0.85, 1.00, 1.15, and 1.30 to γ_*o*_, η_*a*_, *c*_*o*_, κ, δ, and ρ, varying one parameter at a time while holding the others at their baseline values. Two-dimensional response regions evaluate *L*×γ_*o*_ and *c*_*o*_×η_*a*_. The first examines how advance information interacts with organizer-controlled direct mitigation, while the second considers the relationship between organizer effort cost and team capacity-building efficiency. Collectively, these checks assess whether the governance conclusions remain stable under alternative assumptions about intervention effectiveness, institutional burden, preparedness retention, and the evolution of event-level heat-health pressure. To complement these one-way checks, a deterministic 120-point Latin-hypercube experiment jointly varies 12 coefficients over 70%−130% of baseline: organizer direct efficiency, team capacity efficiency, organizer effort cost, preparedness protection, risk dissipation, capacity depreciation, all three running risk weights q_*i*_, and all three terminal risk weights ϕ_*i*_. The symmetric ±30% interval is used as a scenario stress band rather than a statistical confidence interval. Each sampled vector is evaluated for Nash, government-led Stackelberg, and cooperative governance in S3 at *L* = 3 using the same numerical settings; rank correlations are reported as descriptive global-sensitivity diagnostics.

### 4.4 Joint-response surfaces and preparation-window thresholds

Two joint-response experiments examine interactions that cannot be identified through one-way parameter perturbations. Heat-health warning systems depend on advance information as well as institutional arrangements capable of translating alerts into protective action (37). The information–capability surface therefore varies early-warning lead time *L* from 0 to 6 and organizer direct mitigation efficiency γ_*o*_ from 0.12 to 0.28. At each parameter combination, cooperative social gain is calculated as the percentage reduction in aggregate social loss relative to the corresponding Nash outcome. All other parameters are held at their baseline values. The surface examines how the value of coordination changes when additional preparation time is combined with stronger organizer-controlled capacity to reduce event-level heat exposure.

The environmental-threshold experiment varies the temporal structure of S3 by applying multipliers *m*_*H*_ and *m*_σ_, ranging from 0.70 to 1.30, to its peak amplitude and duration parameter, respectively. The baseline heat pressure and peak timing remain unchanged. For each *m*_*H*_, *m*_σ_ combination, the cooperative problem is solved at *L* = 0, 1, …, 6. Provided that the reduction in cooperative social loss between *L* = 0 and *L* = 6 is positive, the effective preparation-window threshold $L_{p}^{†}$ is defined as the smallest interpolated lead time at which the reduction from the *L* = 0 outcome reaches a proportion *p* of that total warning benefit. Linear interpolation is applied between the adjacent lead-time values surrounding the first crossing. The main analysis sets (*p* = 0.80); thresholds based on (*p* = 0.70) and (*p* = 0.90) are also calculated to assess sensitivity to the selected benefit criterion. No monotonicity is imposed on the welfare sequence. If the criterion is crossed more than once, the earliest crossing defines the threshold. Because warning lead time is evaluated on a one-unit grid, the crossing is bracketed by adjacent integer lead-time values and threshold estimates are reported to one decimal place; no finer numerical precision is claimed without a denser-grid or root-finding check.

The resulting $L_{80}^{†}$ is a comparative preparation-window indicator defined on the normalized horizon (*T* = 10). It identifies the first modeled lead time at which 80% of the reduction in cooperative social loss available between (*L* = 0) and (*L* = 6) has been captured. Its numerical value depends on the time normalization, the two comparison endpoints, the selected benefit proportion (*p*), the heat-shock profile, and the institutional parameters. Comparisons of $L_{80}^{†}$ are therefore made across scenarios evaluated under the same numerical design. In this study, the term “threshold” denotes the crossing of a specified proportion of modeled warning benefit. Translation into an operational warning interval requires an empirical mapping to the event calendar, forecast performance, exposure conditions, population vulnerability, and the mobilization times of specific protective measures.

## 5 Results

### 5.1 Dynamic adaptation under alternative governance regimes

Figure 2 traces the model-implied residual heat-risk pressure under the three governance regimes in the baseline S3 scenario with *L* = 3. Before warning arrival, the Nash and government-led Stackelberg trajectories remain close, while residual pressure is already lower under cooperation. Following the information update, the cooperative path initially declines as the revised effort profile strengthens adaptive capacity and subsequently rises from a lower level as the late heat shock develops. Its peak and terminal values remain well below those produced by the two noncooperative regimes. The government-led trajectory lies slightly above the Nash path during the late high-risk phase. Within the specified government-first hierarchy and baseline parameterization, commitment changes the risk trajectory only modestly because the actors retain separate objectives; joint optimization yields a larger reduction in unresolved event-level heat-health pressure.

**Figure 2:**
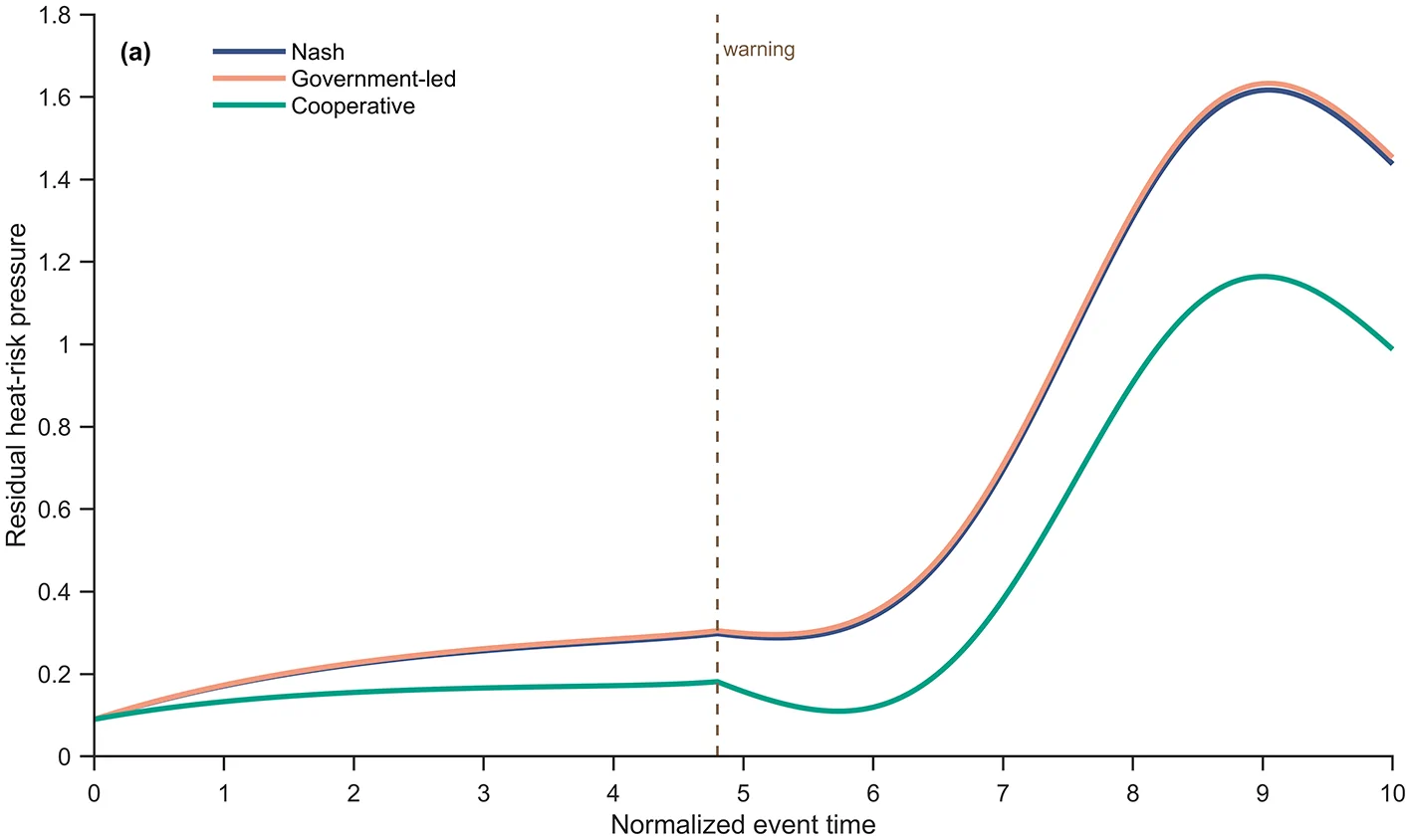
Residual heat-risk pressure under Nash, government-led Stackelberg, and cooperative governance in the baseline S3 scenario with warning lead time *L* = 3. The vertical marker indicates warning arrival.

The effort paths in Figure 3 explain this divergence. Re-optimization at the warning time *t*_*w*_ = 4.8 produces an immediate increase in the controls of all three actors, although the size and composition of the response differ across regimes. Under Nash behavior, team effort remains the largest component, while organizer effort is comparatively limited despite the organizer's relatively effective direct-mitigation channel. Relative to Nash, the baseline government-led Stackelberg solution changes the public authority's effort path but produces only small adjustments in the organizer and team responses. Cooperation increases effort both before and after warning arrival and gives greater weight to organizer-controlled mitigation; during the post-warning period, organizer effort approaches the level supplied by the team. This reallocation is consistent with the shared-state structure of the model. Decentralized organizer effort reflects only the risk reduction valued in the organizer's own objective, whereas cooperation incorporates the effect of that intervention on the losses borne by all three actors.

**Figure 3:**
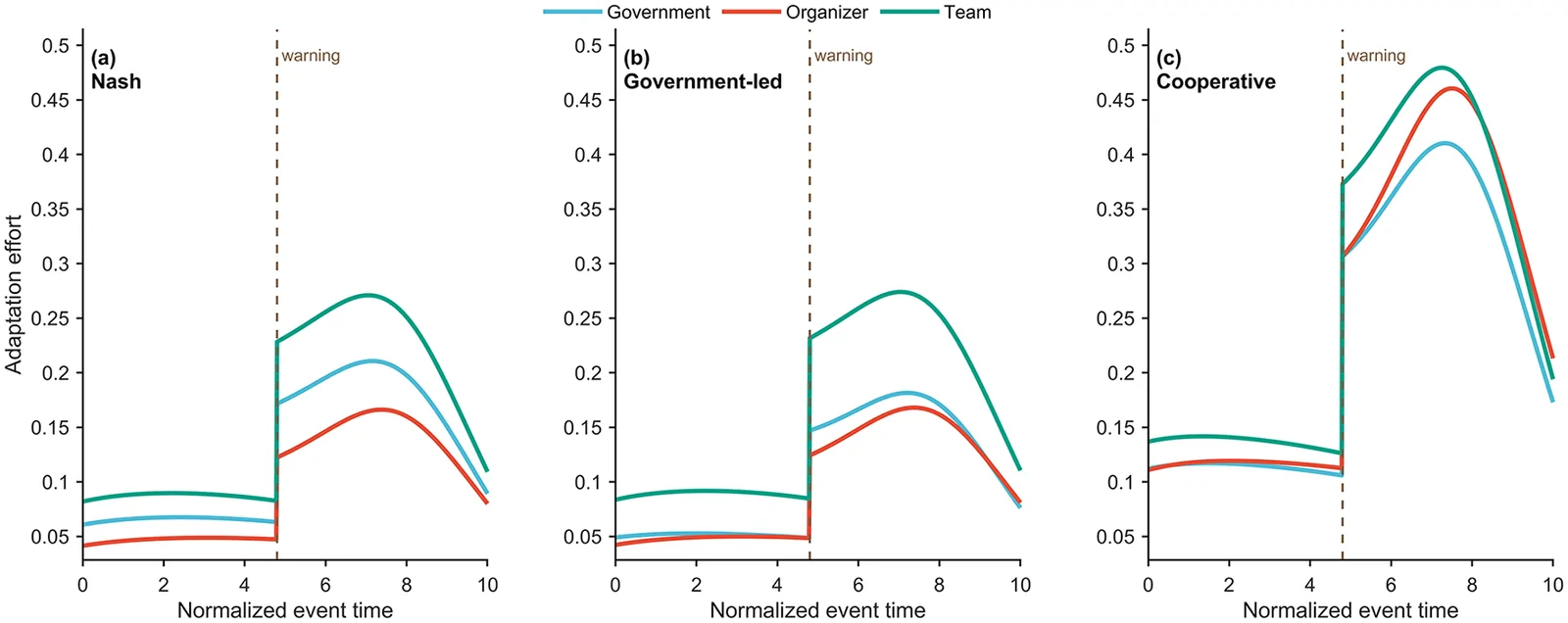
Government, organizer, and team adaptation-effort paths under the three governance regimes. The baseline case uses S3 and *L* = 3. **(a)** Nash, **(b)** Government-led, and **(c)** Cooperative.

Table 3 and Figure 4 quantify the resulting differences. Cooperative social loss is 20.627, compared with 26.418 under Nash and 26.829 under government-led Stackelberg. These values correspond to cooperative reductions of 21.9% and 23.1%, respectively. Peak residual heat-risk pressure decreases from 1.617 under Nash and 1.633 under the government-led regime to 1.164 under cooperation, representing reductions of 28.0% and 28.7%. Terminal residual pressure also falls, from 1.437 and 1.453 under the two noncooperative regimes to 0.988 under cooperation. In the baseline setting, social loss under government-led Stackelberg is 1.6% higher than under Nash. Government cumulative effort decreases from 1.245 under Nash to 1.044 under the government-led regime, whereas organizer and team effort increase only slightly, from 0.958 to 0.972 and from 1.612 to 1.636, respectively. This partial substitution is accompanied by marginally higher peak and terminal residual pressure. The comparison is conditional on the government-first leader assignment, actor-specific objectives, baseline coefficients, and open-loop commitment structure. The reported percentages describe changes in normalized model outcomes; empirical calibration would be required to relate them to heat-related illness, injury, or demand for medical services.

**Table 3:** Baseline outcomes under S3 with *L* = 3.

Outcome	Nash	Government-led	Cooperative
Social loss, *J*_Σ_	26.418	26.829	20.627
Government loss, *J*_*g*_	9.335	9.339	6.649
Organizer loss, *J*_*o*_	6.586	6.742	6.421
Team loss, *J*_*a*_	10.497	10.747	7.557
Peak residual risk	1.617	1.633	1.164
Terminal residual risk	1.437	1.453	0.988
Cumulative government effort	1.245	1.044	2.320
Cumulative organizer effort	0.958	0.972	2.529
Cumulative team effort	1.612	1.636	2.735

Loss and residual-pressure values are normalized model outputs. Losses do not denote monetary amounts, and residual pressure does not denote temperature, a thermal index, or observed health outcomes. Cumulative effort is also expressed in normalized units.

**Figure 4:**
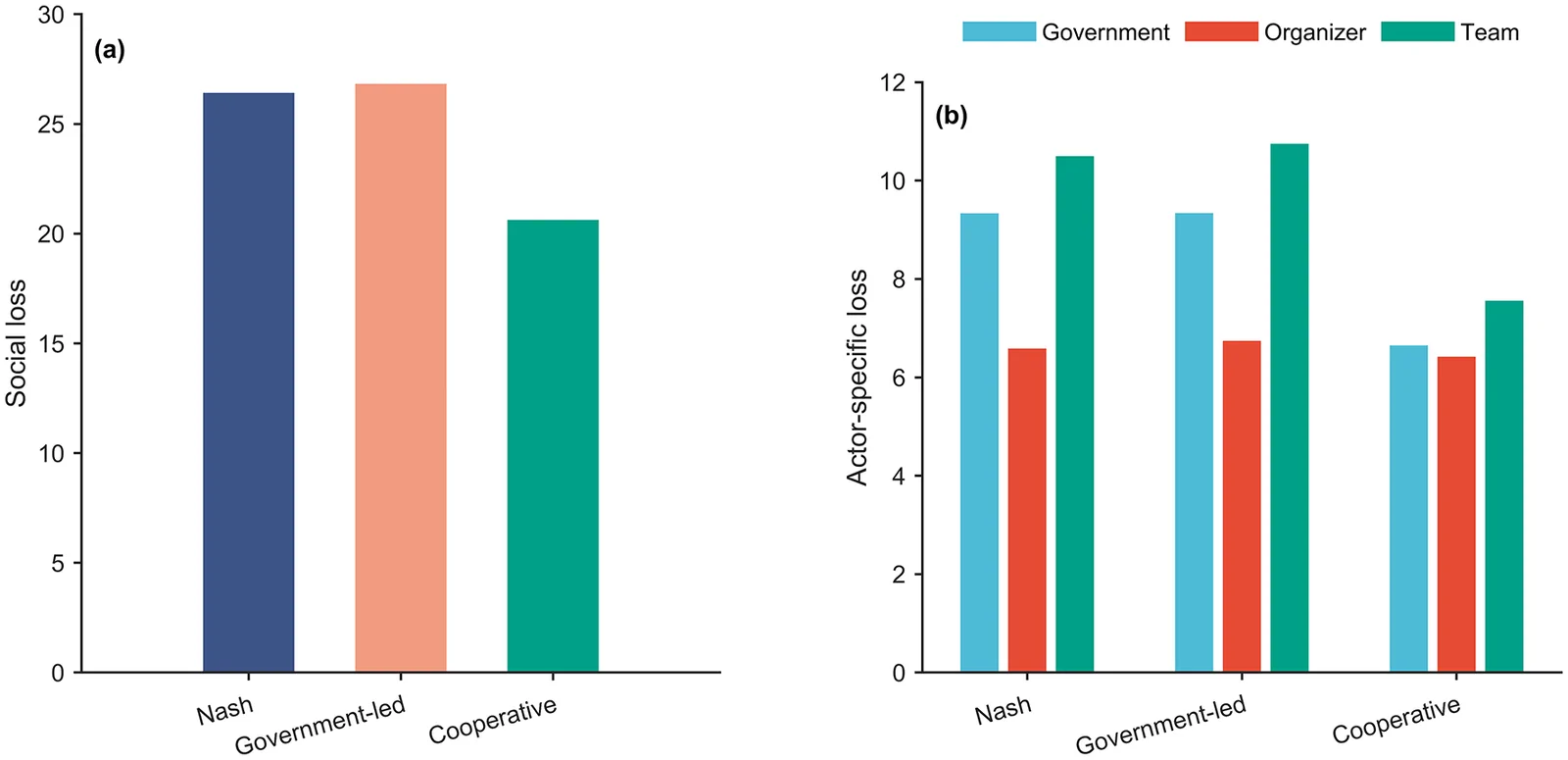
Baseline comparison of normalized aggregate and actor-specific objective values. **(a)** reports aggregate social loss, *J*_Σ_. **(b)** reports the actor-specific objective values under each governance regime. These values have no monetary units.

The actor-specific losses reveal a further distinction between aggregate efficiency and the distribution of cooperative gains. Before contractual redistribution, cooperation lowers the private loss of every actor: government loss decreases from 9.335 to 6.649, team loss from 10.497 to 7.557, and organizer loss from 6.586 to 6.421. The organizer therefore receives the smallest reduction in private loss. At the same time, its cumulative effort rises from 0.958 under Nash to 2.529 under cooperation, the largest proportional increase among the three actors. Although the organizer is not made worse off in the baseline calculation, the allocation asks it to assume a substantially larger role in event-wide protection while yielding only a limited private gain. This divergence between operational responsibility and uncompensated benefit provides the distributional basis for the cost-sharing mechanism examined in Section 5.8.

### 5.2 Robustness to alternative heat-shock structures

Table 4 and Figure 5 show that the governance ordering remains consistent across the four normalized heat-shock scenarios. Cooperation produces the lowest social loss and peak residual heat-risk pressure under S1–S4, while the Nash values remain below those under government-led Stackelberg. Relative to Nash, cooperation reduces social loss by 21.9%−23.2% and peak residual pressure by 28.0%−33.5% across the four scenarios. Social loss under the government-led regime exceeds Nash by 1.6%−2.0%. Outcome levels vary with the heat profile, while the ordering remains stable across the four scenarios considered.

**Table 4:** Social loss and peak residual heat-risk pressure across heat scenarios.

Scenario	Nash social loss	Government-led social loss	Cooperative social loss	Nash peak risk	Government-led peak risk	Cooperative peak risk
S1	31.538	32.176	24.229	1.516	1.536	1.043
S2	62.587	63.845	48.090	1.957	1.987	1.302
S3	26.418	26.829	20.627	1.617	1.633	1.164
S4	29.467	29.939	22.861	1.415	1.431	0.987

**Figure 5:**
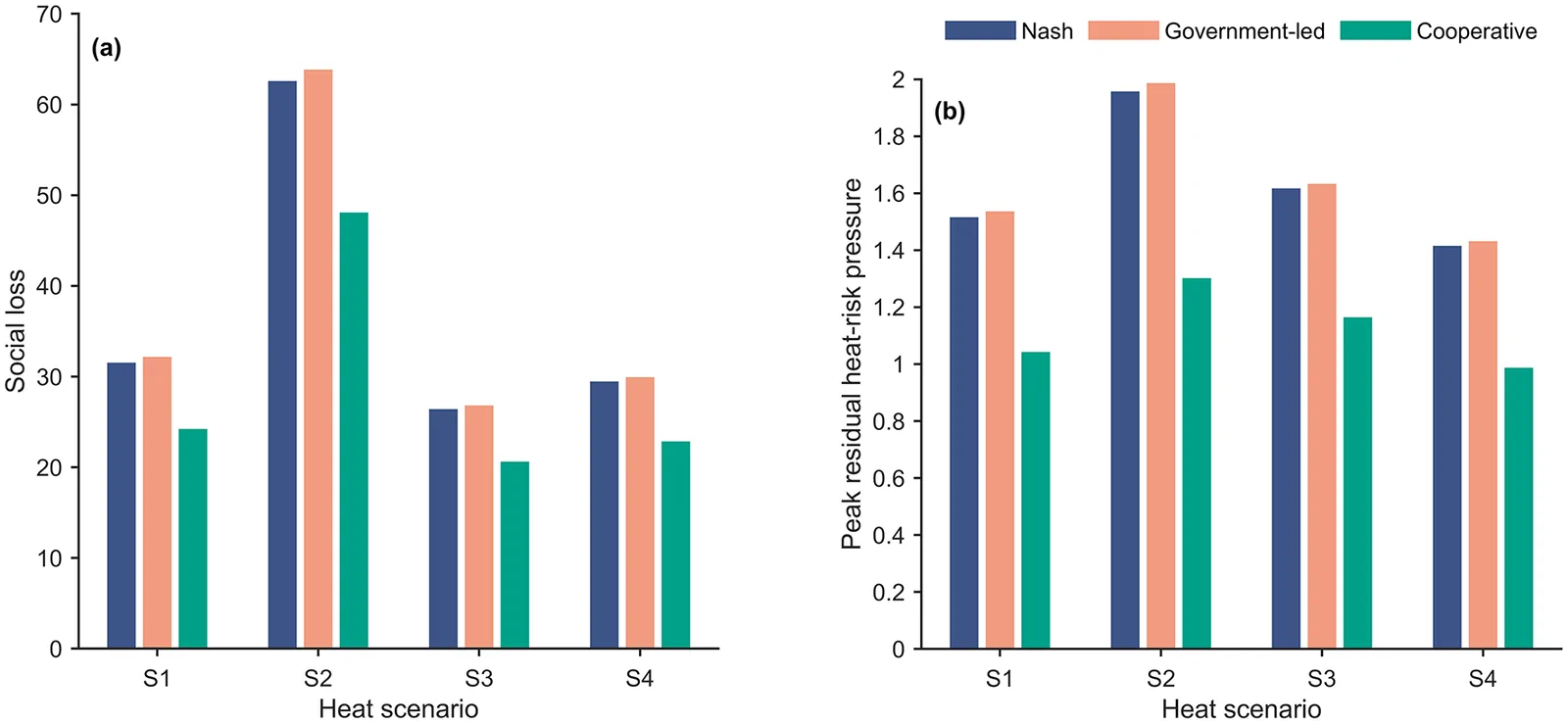
Governance-regime comparison across four normalized heat-shock structures. **(a)** reports social loss and **(b)** peak residual heat-risk pressure.

S2 produces the largest social loss and peak residual pressure under every regime. This scenario combines the highest baseline heat pressure with the broadest single peak, keeping external forcing elevated over a larger part of the finite horizon. Sustained heat therefore requires protective effort and adaptive capacity to be maintained for longer, increasing both the cumulative residual-risk component and the effort-cost component of social loss. Because S2 changes several heat-shock characteristics simultaneously, its comparison with the other scenarios does not isolate a pure duration effect. The separate preparation-window response surface reported later examines the roles of heat-shock intensity and persistence more directly.

The double-peak structure in S4 provides a different test of temporal robustness. Under all three regimes, S4 generates greater social loss than S3 even though its peak residual pressure is lower. Repeated moderate pressure can therefore produce a larger horizon-wide loss without generating the highest instantaneous risk value. This distinction is relevant to event heat-health management because a single peak indicator may not capture the demands created by separated periods of elevated risk and the need to maintain preparedness between them. The cooperative advantage remains under this recurrent forcing pattern, showing that the baseline ordering is not confined to the late single-peak structure of S3. This result is conditional on the government-first leader assignment, specified state dynamics, common information structure, and normalized parameterization; alternative forecast-error structures, capacity constraints, or intervention dynamics may change the ranking.

### 5.3 Value of early-warning lead time

Figure 6 and Table 5 report the response of social loss and peak residual heat-risk pressure as warning lead time increases from *L* = 0 to *L* = 6 under S3. The physical heat-shock path is held constant, so the differences arise from the time available to revise mitigation and preparedness decisions. In the L=0 case, event-specific information arrives at the reference heat peak; actors can still revise subsequent actions, but no pre-peak preparation window is available. Both outcomes decline monotonically with lead time under all three governance regimes. Nash social loss falls from 31.064 to 25.154, a reduction of 19.0%, while social loss under government-led Stackelberg decreases from 31.385 to 25.652, or 18.3%. Under cooperation, the corresponding decline is from 25.290 to 20.017, equivalent to 20.8%.

**Figure 6:**
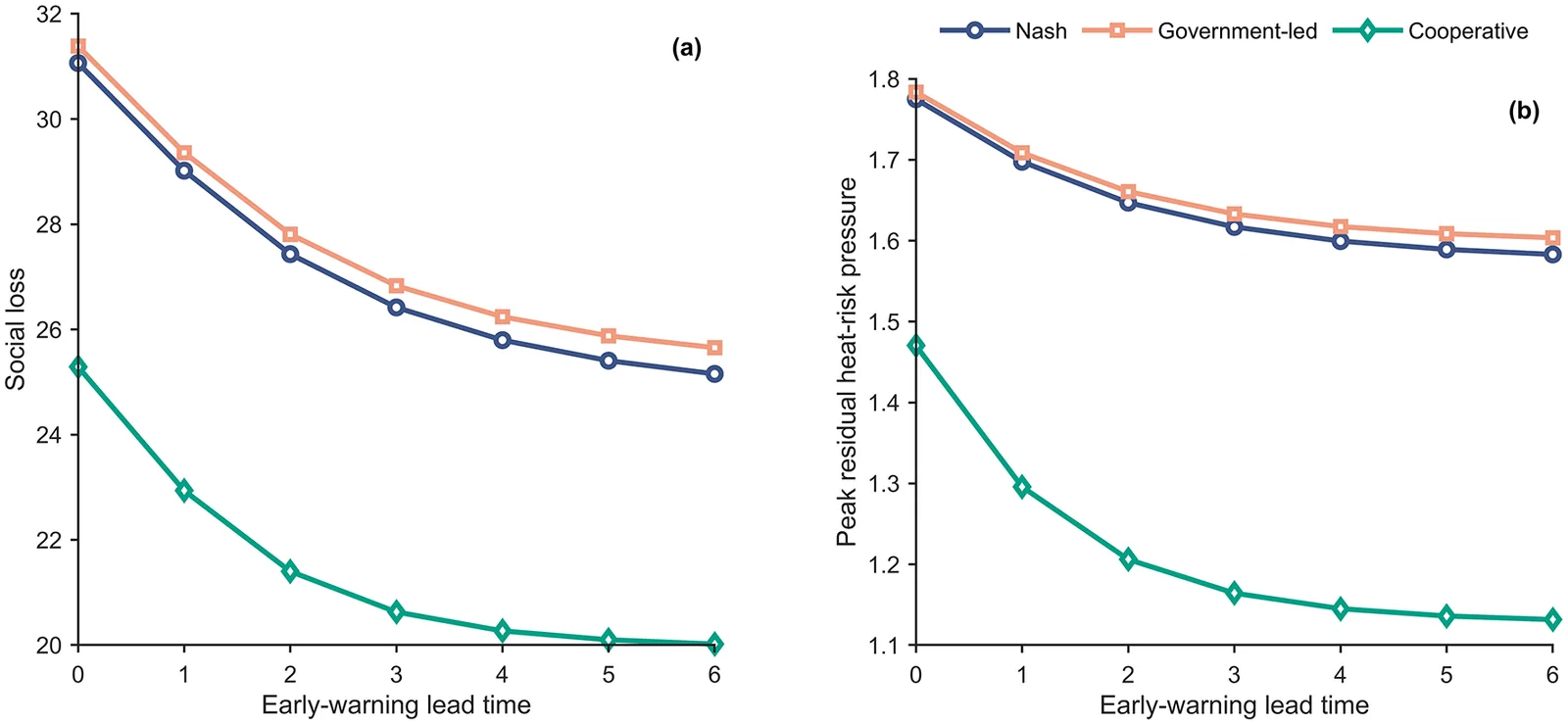
Early-warning lead-time response under S3. Panel **(a)** shows social loss and panel **(b)** peak residual heat-risk pressure for *L* = 0, …, 6.

**Table 5:** Early-warning experiment under S3.

*L*	Nash social loss	Government-led social loss	Cooperative social loss	Nash peak risk	Government-led peak risk	Cooperative peak risk
0	31.064	31.385	25.290	1.775	1.784	1.470
1	29.017	29.360	22.936	1.698	1.709	1.296
2	27.431	27.808	21.401	1.647	1.661	1.206
3	26.418	26.829	20.627	1.617	1.633	1.164
4	25.796	26.239	20.268	1.600	1.617	1.145
5	25.405	25.878	20.099	1.589	1.609	1.136
6	25.154	25.652	20.017	1.583	1.604	1.132

The peak-risk results reveal a larger difference in how the regimes use advance information. Peak residual pressure decreases by 10.8% under Nash and 10.1% under the government-led regime, compared with 23.0% under cooperation. Cooperation also retains the lowest social loss and peak residual pressure at every value of L. Within the model, joint planning converts additional lead time into stronger pre-peak protection because the benefits of immediate mitigation and accumulated adaptive capacity are evaluated across all three actor objectives. Under the specified government-first hierarchy and parameterization, the government-led response remains close to Nash because the organizer and team continue to optimize actor-specific objectives. For event heat-health protection, the comparison suggests that the availability of earlier information and the institutional capacity to coordinate action are jointly relevant to controlling the maximum unresolved risk pressure.

The gains are concentrated in the earlier part of the preparation window. For Nash, increasing lead time from *L* = 0 to *L* = 1 reduces social loss by 2.047, while the reduction from *L* = 5 to *L* = 6 is 0.251. The corresponding changes are 2.025 and 0.226 under the government-led regime, and 2.354 and 0.082 under cooperation. By *L* = 3, the three regimes have captured 78.6%, 79.5%, and 88.4%, respectively, of the total social-loss reduction available between L = 0 and L = 6. The progressively flatter response indicates diminishing marginal value as the preparation window expands: once the principal adjustments in effort timing and adaptive capacity have been made, each additional unit of lead time produces a smaller modeled improvement. This pattern does not identify a universal operational warning period, because *L* remains expressed in normalized event time and the experiment assumes accurate, simultaneous information uptake by all actors.

### 5.4 How early warning changes adaptation timing

Figure 7 examines how warning lead time affects actor-specific effort under cooperative governance in the S3 scenario. Panel (a) shows a monotonic decline in peak effort for the government, organizer, and team as *L* increases. The largest decreases occur over the earlier lead-time increments, after which the curves gradually flatten. Team effort retains the highest peak throughout the experiment, followed by organizer and government effort. When information arrives close to the heat peak, public-health preparedness, event-level mitigation, and team-support actions must be concentrated within a short interval. An earlier update allows these actions to be planned over a broader preparation window, reducing the maximum modeled effort required at any single point. In operational terms, the result indicates a less compressed response burden, although the normalized controls do not measure actual staffing, expenditure, or medical-service capacity.

**Figure 7:**
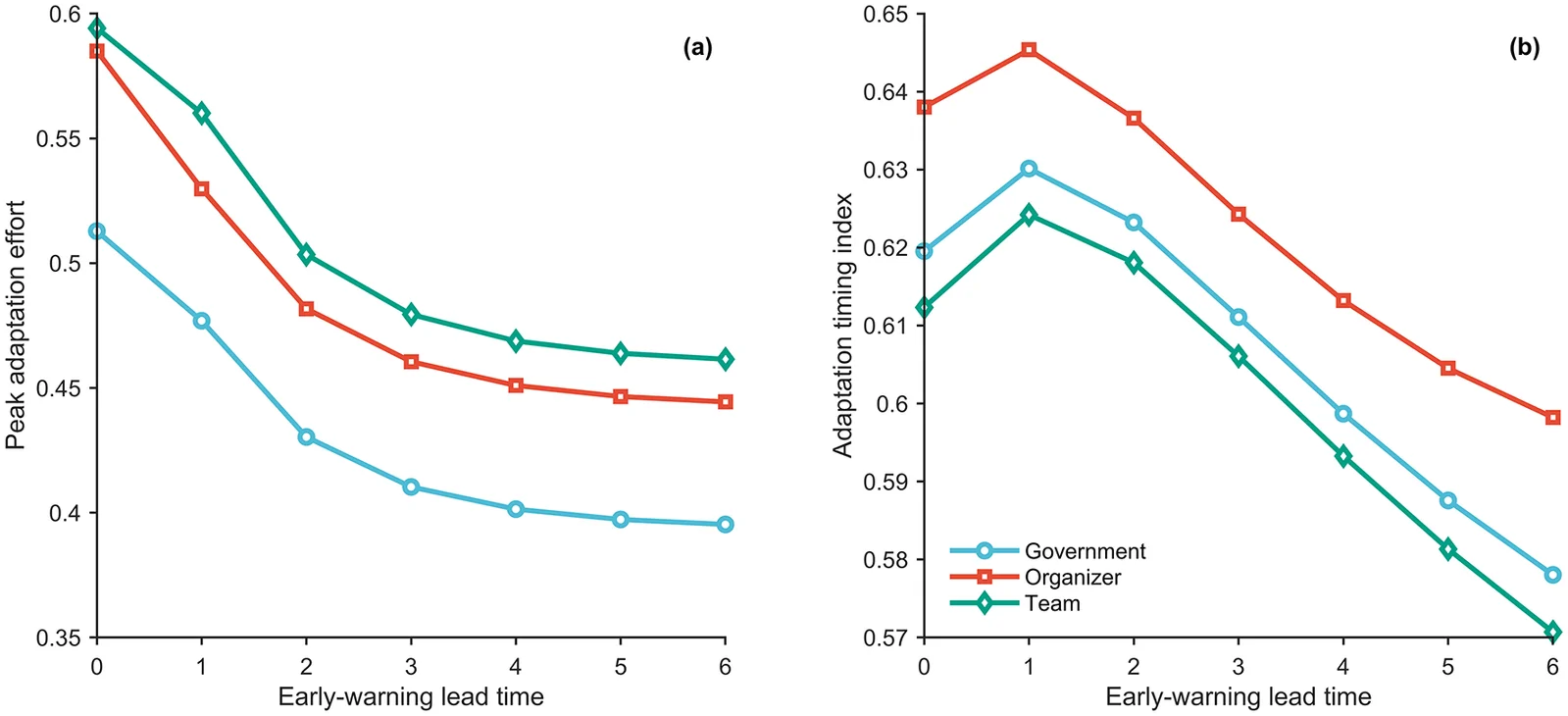
Effect of early-warning lead time on actor-specific adaptation intensity and timing under cooperative governance in the S3 scenario. Panel **(a)** reports peak actor effort. Panel **(b)** reports the Adaptation Timing Index, where lower values indicate an earlier effort-weighted temporal center.

The Adaptation Timing Index in Figure 7b reveals a more nuanced temporal adjustment. Because a lower *ATI*_*i*_ indicates an earlier effort-weighted center, the small increase from *L* = 0 to *L* = 1 means that the first unit of advance warning reduces peak effort without immediately shifting the overall center of action toward the beginning of the horizon. At this short lead time, the revised controls remain concentrated relatively close to the high-risk period. From *L* = 1 onward, the index declines steadily for all three actors, showing that a longer preparation window permits a larger share of effort to be supplied earlier. By *L* = 6, the team has the earliest temporal center, the government occupies an intermediate position, and organizer effort remains comparatively later. This ordering is consistent with the team's stronger capacity-building channel and the organizer's greater role in event-proximate direct mitigation.

Taken together, the two panels identify a staged timing response. Limited advance information primarily reduces the intensity of the effort peak, while a sufficiently long preparation window also moves the center of protective action earlier. The external heat-shock path remains fixed throughout the comparison. Improvements in social loss and residual risk therefore arise from earlier re-optimization and temporal redistribution of effort, with no assumed reduction in heat severity. Early warning functions here as a governance resource that expands the time available to coordinate health protection. The result remains conditional on accurate and simultaneous information arrival and does not establish an operational warning period for a specific event.

### 5.5 Responsibility reallocation under cooperation

Figure 8 compares actor-specific cumulative effort and responsibility shares in the baseline S3 scenario with *L* = 3. Under Nash, the team supplies 42.3% of total effort, followed by the government at 32.6% and the organizer at 25.1%. The government-led Stackelberg regime changes this composition only moderately: the team share rises to 44.8%, the organizer's share to 26.6%, and the government share falls to 28.6%. Cooperation produces a more balanced allocation, with shares of 36.1% for the team, 33.3% for the organizer, and 30.6% for the government. Relative to Nash, the organizer gains 8.2 percentage points of responsibility, while the team and government shares decline by 6.2 and 2.0 percentage points, respectively.

**Figure 8:**
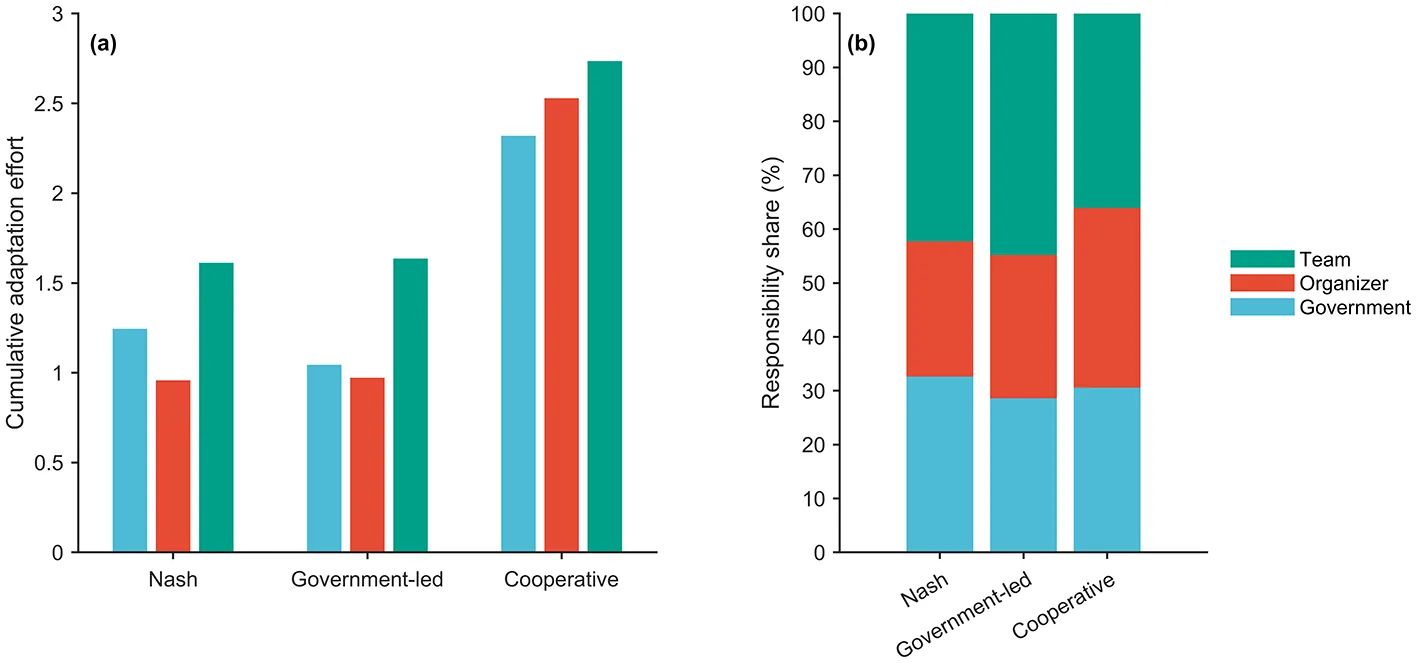
Actor-specific cumulative adaptation effort and responsibility shares under Nash, government-led Stackelberg, and cooperative governance in the baseline S3 scenario with *L* = 3. Panel **(a)** reports cumulative effort. Panel **(b)** reports responsibility shares, which sum to 100% within each regime.

These relative changes occur alongside a substantial expansion in absolute effort. Total cumulative effort rises from 3.815 under Nash to 7.584 under cooperation, an increase of 98.8%. Government effort increases from 1.245 to 2.320, organizer effort from 0.958 to 2.529, and team effort from 1.612 to 2.735. The lower cooperative share assigned to the team therefore does not represent a reduction in team involvement; the team supplies more effort in absolute terms while accounting for a smaller proportion of the expanded joint response. The organizer experiences the largest proportional increase, consistent with the shift toward event-level mitigation identified in Section 5.1. Cooperation thus changes both the scale and composition of adaptation instead of applying a uniform increase to the Nash effort profile.

Within the normalized parameterization, this reallocation reflects the relative effectiveness and cost of the available control channels. The organizer has the highest direct mitigation coefficient, γ_*o*_ = 0.20, compared with γ_*g*_ = 0.08 and γ_*a*_ = 0.05. Once the effects of organizer-controlled action on all three objectives are incorporated, this direct channel is used more intensively despite the organizer's relatively high effort-cost coefficient. The resulting allocation also depends on capacity-building efficiencies, state persistence, and actor-specific loss weights, so the 33.3% organizer share is a conditional model result rather than a prescribed division of institutional duties. In public-health terms, cooperation places greater emphasis on interventions with event-wide reach while retaining substantial contributions from public-health preparedness and team-level protection. Implementing such an allocation would still require authority, resources, and financing arrangements that correspond to the additional responsibilities assigned to each institution.

### 5.6 Parameter sensitivity and two-dimensional response regions

Figures 9, 10 show that the ordering of the three governance regimes is unchanged across the one-way parameter tests. Cooperative governance consistently produces the lowest social loss and peak residual heat-risk pressure, while Nash remains slightly below government-led Stackelberg throughout the tested one-way parameter ranges. Both outcomes decline as organizer direct mitigation efficiency γ_*o*_, team adaptive-capacity efficiency η_*a*_, the protective effect of adaptive capacity κ, or the dissipation rate of gross risk pressure δ increases. Higher organizer effort cost *c*_*o*_ and faster adaptive-capacity depreciation ρ lead to higher losses and peak pressure. The responses are most pronounced for δ and κ. Within the model, the persistence of unresolved heat pressure and the protective contribution of accumulated preparedness therefore have a particularly strong influence on event-level health protection. Although outcome levels vary across the tested multipliers, cooperation remains clearly separated from the two decentralized regimes in every panel. The joint experiment gives the same conditional ordering in all 120 completed samples. Cooperation has the lowest aggregate loss in 120/120 samples, and its reduction relative to Nash ranges from 14.2 to 27.9%. Government-led Stackelberg does not fall below Nash in any sampled vector; its median social loss is 1.6% higher than Nash. The largest absolute rank correlations with the cooperative-gain percentage are associated with preparedness protection (κ, 0.60) and gross-risk dissipation (δ, −0.49), while the q_*i*_ and ϕ_*i*_ weights are included in the joint uncertainty set rather than held fixed.

**Figure 9:**
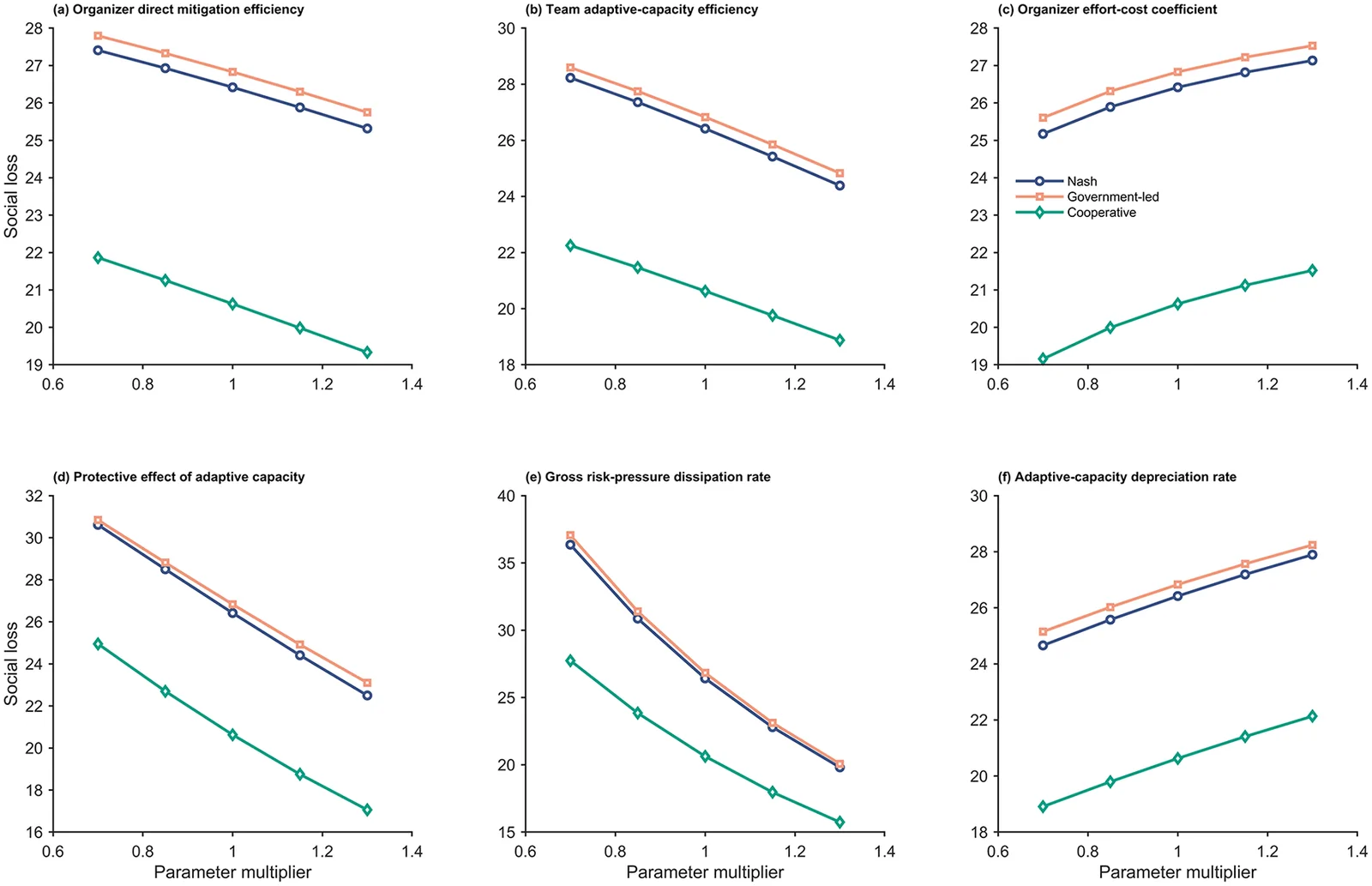
One-way parameter sensitivity of social loss. Each parameter is multiplied by 0.70, 0.85, 1.00, 1.15, and 1.30 while the remaining baseline parameters are held fixed.

**Figure 10:**
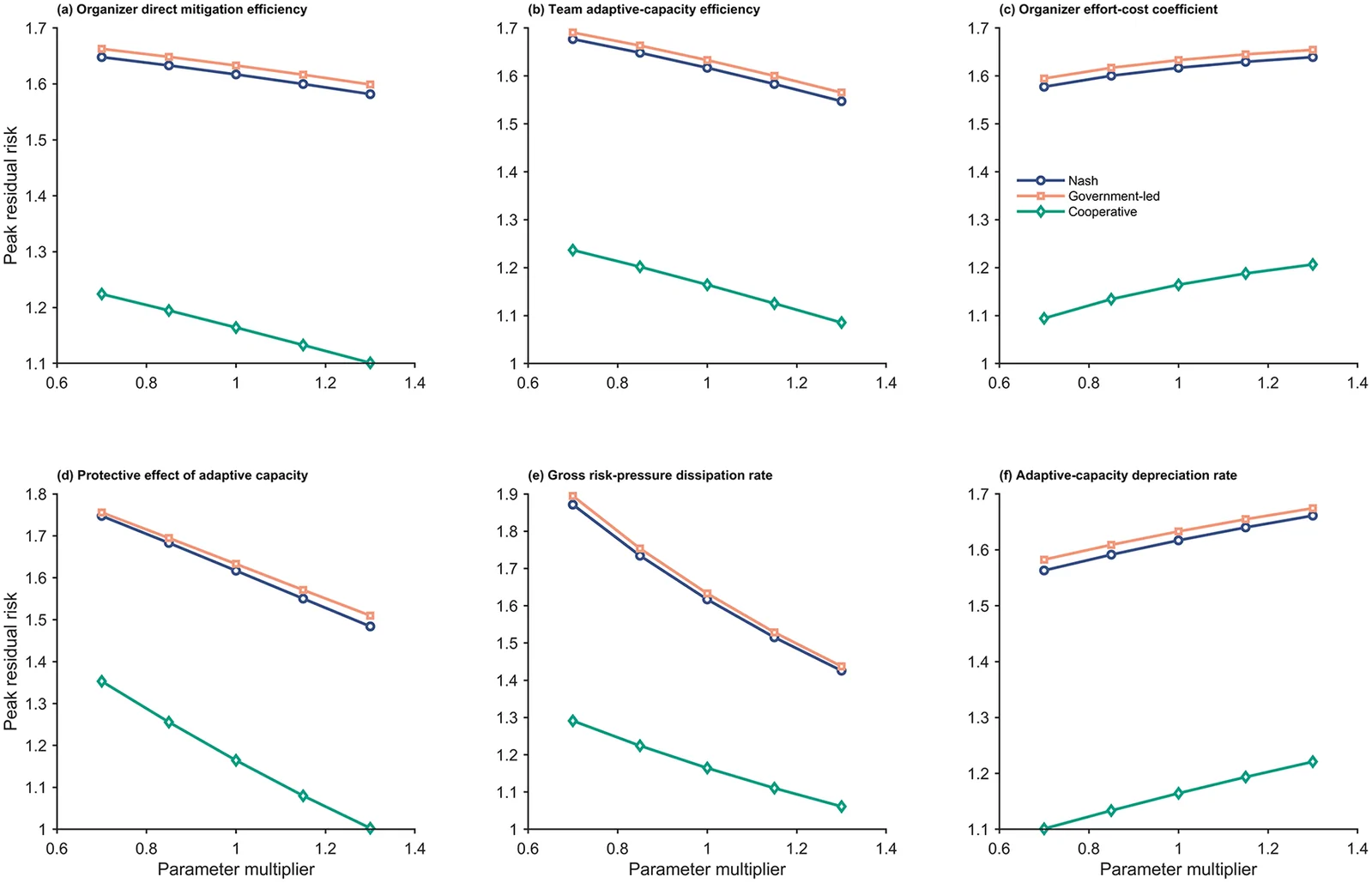
One-way parameter sensitivity of peak residual heat-risk pressure under the same parameter multipliers.

Figure 11 considers warning lead time and organizer direct mitigation efficiency jointly. Cooperative social gain and peak-risk reduction remain positive throughout the response regions. At a given lead time, stronger organizer efficiency increases the advantage of cooperation by improving a control channel that acts directly on shared event exposure. The pattern along the lead-time dimension differs between the two outcomes. Social gain is strongest at short to intermediate preparation windows and then gradually narrows, while peak-risk reduction increases with additional lead time before approaching a plateau. Earlier information therefore contributes to cumulative welfare and acute peak control in different ways. Its protective value also depends on whether the organizer has sufficient capability to translate warning information into changes in event operations. Figure 12 presents the same information–capability relationship in three-dimensional form.

**Figure 11:**
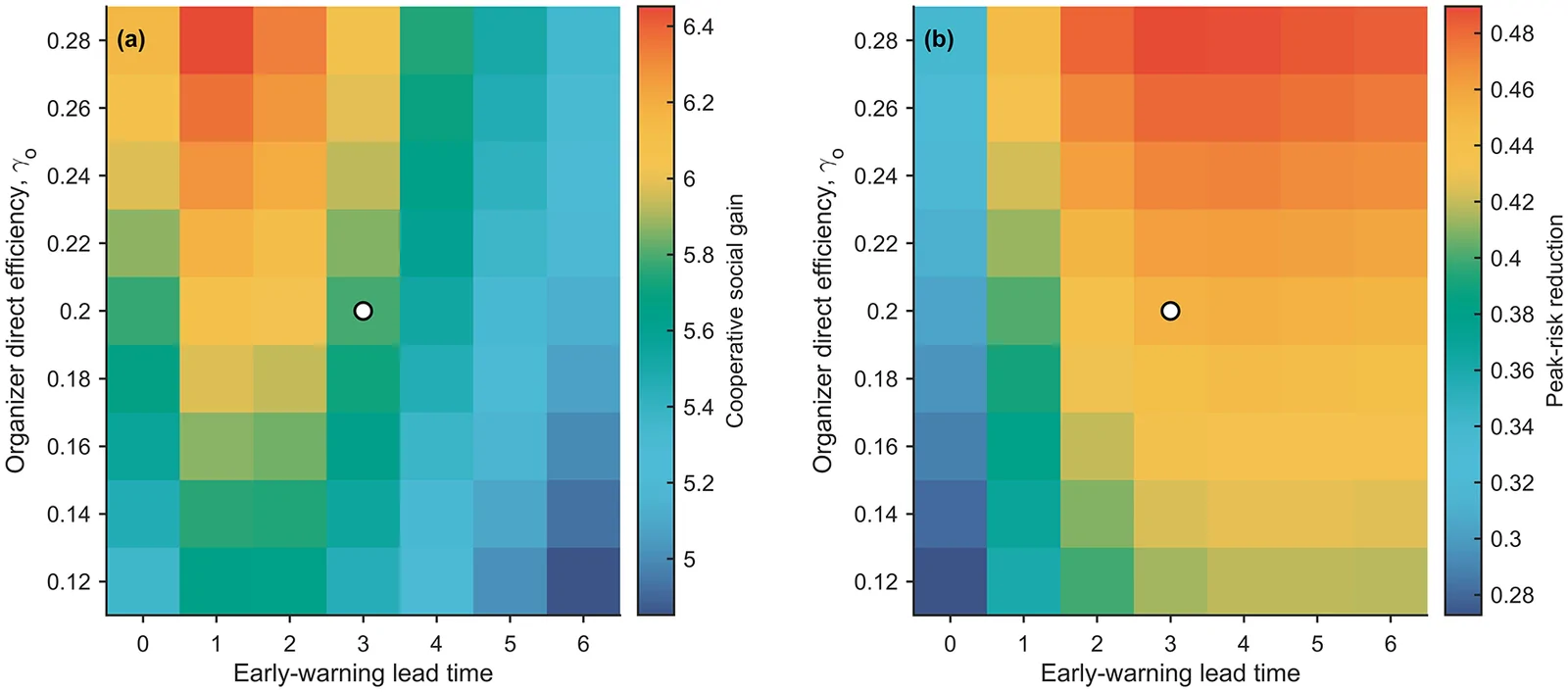
Cooperative response regions over early-warning lead time and organizer direct mitigation efficiency. Panel **(a)** reports cooperative social gain relative to Nash. Panel **(b)** reports peak-risk reduction. The white marker denotes the baseline parameter combination.

**Figure 12:**
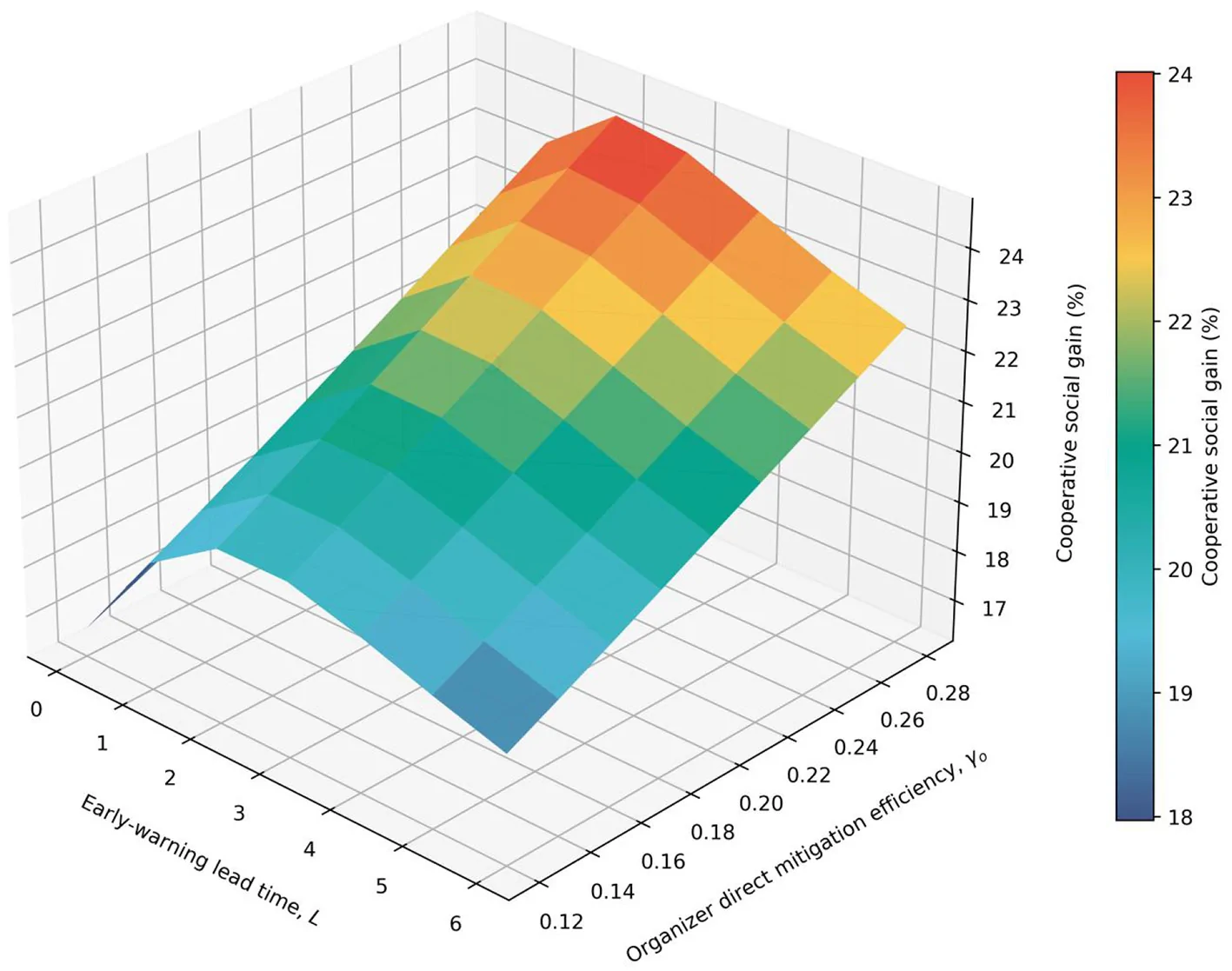
Joint response surface of cooperative social gain over early-warning lead time and organizer direct mitigation efficiency. Cooperative social gain is the percentage reduction in social loss relative to Nash at the same parameter combination. The white marker denotes the baseline (*L* = 3, γ_*o*_ = 0.20).

Figure 13 examines the interaction between organizer effort cost and team adaptive-capacity efficiency. The cooperative advantage remains positive across the full parameter grid, although it becomes smaller as organizer effort grows more expensive. Greater team capacity efficiency strengthens the persistent protection channel and supports peak-risk control when direct organizer action is costly. Because a more effective team channel also improves the Nash outcome, the comparative social gain from cooperation does not necessarily increase with η_*a*_. An improvement in a protective channel can therefore lower overall heat-health risk while narrowing the difference between governance regimes. Peak-risk reduction nevertheless remains positive under every tested combination of *c*_*o*_ and η_*a*_.

**Figure 13:**
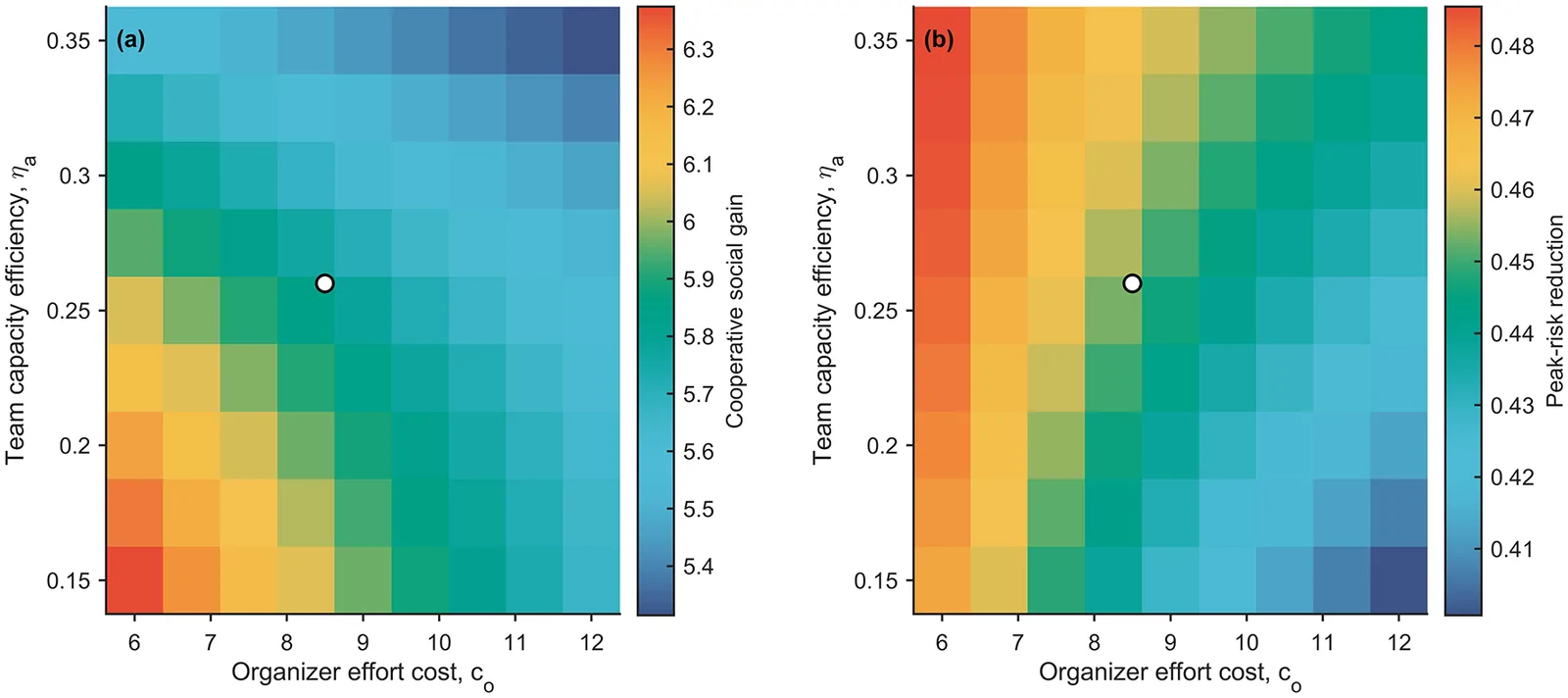
Cooperative response regions over organizer effort cost and team adaptive-capacity efficiency. Panel **(a)** reports cooperative social gain relative to Nash. Panel **(b)** reports peak-risk reduction. The white marker denotes the baseline.

The sensitivity analysis supports the stability of the central coordination result within the normalized ranges considered. Empirical values for intervention effectiveness, preparedness retention, and institutional cost remain to be established; a different leader assignment, alternative state dynamics, binding resource constraints, additional strategic actors, discrete suspension decisions, or forecast error could change the ordering. The response regions describe interactions within the model and provide conditional evidence about the organization of event heat-health protection rather than clinical thresholds or operational rules for a particular sporting event.

### 5.7 Information–capability interaction and the effective preparation window

Figure 12 places warning lead time and organizer direct mitigation efficiency on a common response surface. Cooperative social gain is positive across the full domain, ranging from 16.73 to 24.40%. At the baseline combination of *L* = 3 and γ_*o*_ = 0.20, the gain is 21.92%. The surface rises with organizer efficiency at every value of L, indicating that coordination becomes more valuable when organizers can translate joint decisions into effective changes in event exposure. The response to lead time follows a different pattern. Relative gain rises sharply from *L* = 0, reaches its highest region around (*L* = 2–3), and then declines moderately. Social loss continues to fall as the warning arrives earlier, as shown in Figure 6, but Nash also benefits from the longer preparation window. The percentage advantage of cooperation is therefore greatest after enough time has become available for coordinated preparation, before decentralized adjustment captures a larger share of the information benefit.

Figure 14 examines the preparation time needed to realize most of the cooperative warning benefit under different heat-shock conditions. Across intensity and duration multipliers of 0.70–1.30, $L_{80}^{†}$ ranges from 2.2 to 2.6 normalized time units, with a baseline value of 2.4. Most of this variation is associated with heat-shock duration. When intensity remains at its baseline level, increasing the duration multiplier from 0.70 to 1.30 raises $L_{80}^{†}$ from 2.2 to 2.6. When duration is fixed at baseline, changing the intensity multiplier over the same range moves the threshold only from 2.4 to 2.4. The alternative benefit criteria produce the same pattern. $L_{70}^{†}$ ranges from 1.7 to 2.0, while $L_{90}^{†}$ ranges from 3.0 to 3.5. Social loss declines monotonically with lead time at every tested environmental combination, so each threshold is obtained from a unique first crossing of its respective benefit criterion.

**Figure 14:**
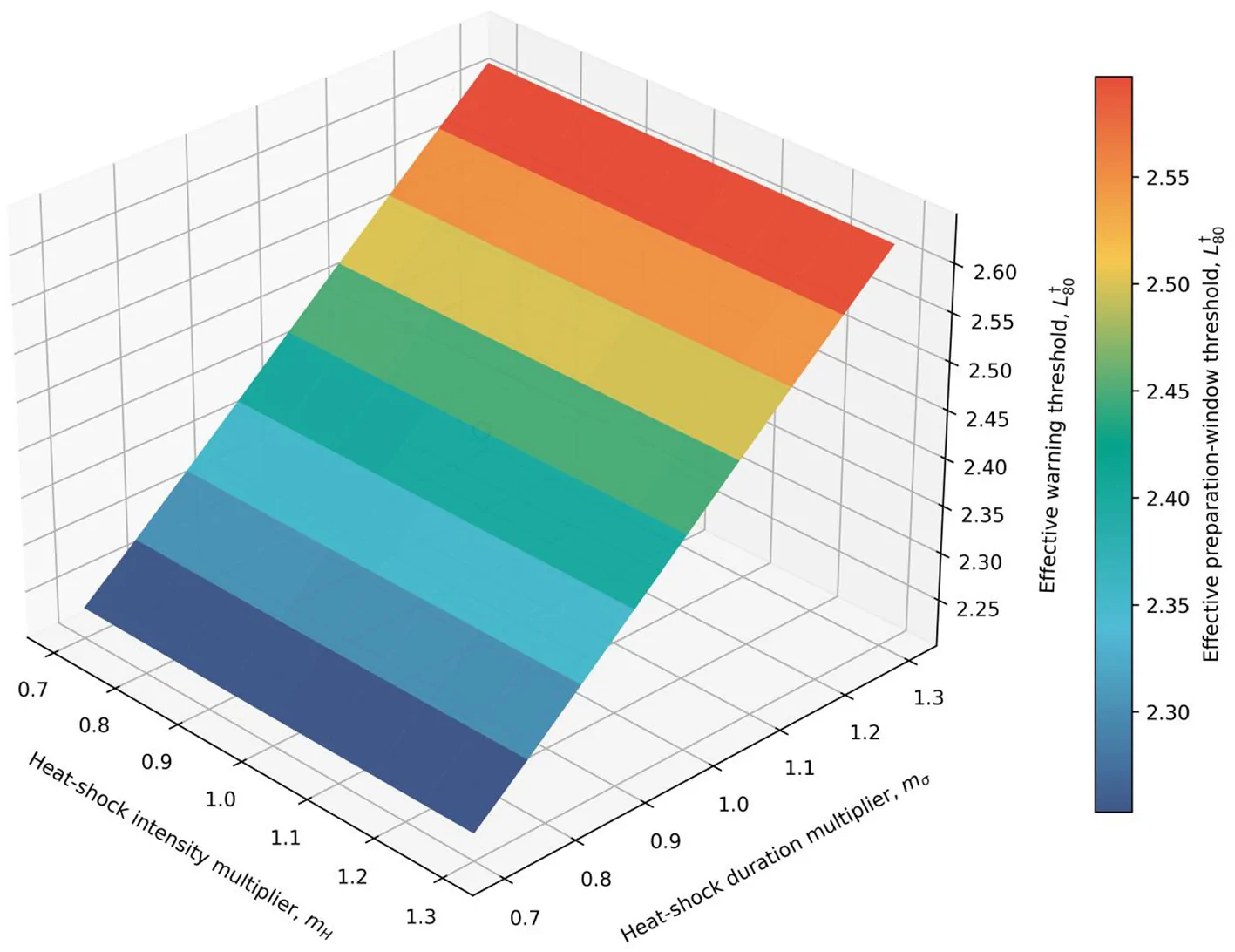
Comparative effective preparation-window threshold across normalized heat-shock intensity and duration multipliers. $L_{80}^{†}$ is the first normalized lead time at which 80% of the cooperative warning benefit available between (*L* = 0) and (*L* = 6) is captured. Values indicate relative positions within the modeled event horizon and support comparisons across parameter combinations. The white marker denotes the baseline environmental combination (*m*_*H*_ = 1, *m*_σ_ = 1). Threshold values are interpreted at one-decimal resolution because the underlying lead-time grid has unit spacing.

The preparation-window surface shows how the modeled lead time required to capture 80% of the available cooperative warning benefit changes with heat-shock intensity and duration. Greater intensity increases the total reduction in modeled loss that can be achieved through earlier warning, while exerting little influence on the fraction of that benefit captured at a given lead time. Duration has a stronger effect because persistent forcing occupies a larger part of the event horizon and extends the period over which adaptive capacity must be maintained. The values from 2.2 to 2.6 identify relative positions within the normalized horizon (*T* = 10). Their comparative ordering indicates that a longer-duration heat shock requires preparation to begin earlier in the modeled sequence. An event-specific interval in hours or days would need to be estimated from the actual competition schedule, forecast horizon, authorization procedures, and implementation time required for the relevant protective measures.

### 5.8 Decentralizing cooperation through cost sharing

Table 6 and Figure 15 show that the baseline cooperative allocation satisfies individual rationality before transfers are introduced: ($J_{i}^{C}<J_{i}^{N}$) for all (*i* ∈ *g, o, a*). Relative to Nash, cooperation lowers the government's and team's objective values by 2.686 and 2.939 normalized loss units, respectively, and the organizer's by 0.165. The organizer therefore receives the smallest model-implied gain while its share of adaptation effort increases substantially, creating a mismatch between operational responsibility and the distribution of the cooperative surplus.

**Table 6:** Baseline cost-sharing coordination results.

Actor	Nash loss	Original cooperative loss	Contracted cooperative loss	Net transfer	Participation gain
Government	9.335	6.649	7.405	0.756	1.930
Organizer	6.586	6.421	4.656	−1.765	1.930
Team	10.497	7.557	8.567	1.009	1.930

Positive transfer denotes a net payer; negative transfer denotes compensation.

All losses, participation gains, and transfers are expressed in normalized loss units. The transfers are model-based settlement values and do not represent currency amounts.

**Figure 15:**
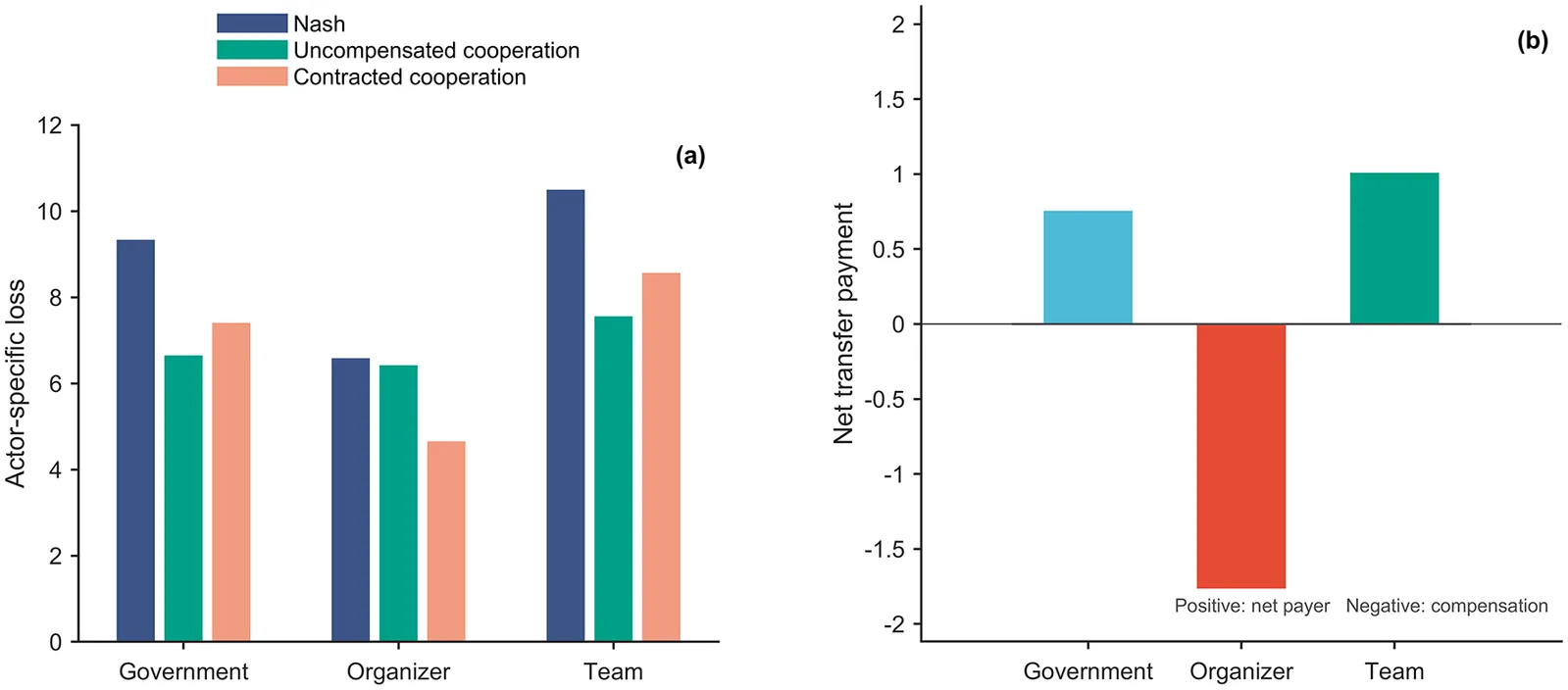
Cost-sharing coordination in the baseline case. Panel **(a)** compares Nash, uncompensated cooperation, and contracted cooperation at actor level. Panel **(b)** reports budget-balanced net transfers. All values are expressed in normalized loss units; the transfers are not monetary payments.

Under the equal-sharing specification, (θ_*i*_ = ω_*i*_ = 1/3), the cooperative surplus is divided equally while the marginal incentives supporting the cooperative effort path are retained. The contracted losses are 7.405 for government, 4.656 for the organizer, and 8.567 for the team. Government makes a net payment of 0.756 and the team pays 1.009, while the organizer receives compensation of 1.765. These transfers are budget balanced. After redistribution, each actor remains better off than under Nash and receives the same participation gain of 1.930.

The two components of the contract perform different roles. The term θ_*i*_*J*_Σ_ links each actor's marginal decision to the aggregate loss generated by the shared heat-risk state. The fixed term *K*_*i*_ determines how the cooperative surplus is distributed without altering those marginal incentives. The organizer's negative transfer in Figure 15b compensates for the additional responsibility assigned to an institution whose direct private benefit from cooperation is comparatively small. In the baseline case, compensation is therefore used to address the distribution of cooperative gains, not to restore participation.

From a public-health perspective, this arrangement illustrates how interinstitutional financing can support event-wide heat protection when the institution controlling important exposure-management measures bears operational costs while the resulting health benefits extend across authorities, teams, and event participants. The reported transfers remain expressed in normalized loss units rather than monetary values. Translating this mechanism into an operational funding agreement would require empirical valuation, clearly defined settlement rules, and an enforceable allocation of responsibilities.

## 6 Discussion

### 6.1 Government leadership under actor-specific objectives

Across the four heat-shock structures, the simulations yield ($J_{\Sigma }^{C}<J_{\Sigma }^{N}<J_{\Sigma }^{S}$). The lower cooperative value follows from the construction of the benchmark, which minimizes *J*_Σ_ over the same admissible controls. The Nash–Stackelberg ordering is an equilibrium result. In the baseline S3 case with (*L* = 3), government-led Stackelberg governance produces slightly higher social loss than Nash, and the same direction is retained across S1–S4 and the reported one-way parameter tests. These comparisons establish robustness within the tested normalized design. Their magnitude and direction remain linked to the leader assignment, objective weights, intervention efficiencies, effort costs, and state dynamics. The Stackelberg results therefore characterize the government-first hierarchy specified in Section 3.5; organizer- and team-led hierarchies remain separate cases. No reversal of the Nash–government-led Stackelberg ordering was observed across S1–S4, the reported one-way parameter ranges, or the 120-point joint sensitivity design; this is a numerical finding for the tested design rather than a general property of Stackelberg leadership.

The mechanism follows from the criterion used by the government leader. The government anticipates the organizer's and team's responses while continuing to minimize *J*_*g*_, whereas aggregate performance is evaluated through *J*_Σ_. In the baseline solution, government cumulative effort declines relative to Nash, while the corresponding increases in organizer and team effort are modest. This partial substitution leaves peak and terminal residual pressure slightly higher. Government commitment consequently changes the strategic response without fully incorporating the losses represented in the organizer's and team's objectives.

The government-first specification captures the public authority's event-wide health-protection, resource-commitment, and cross-agency coordination functions, while scheduling and venue controls remain assigned to the organizer. Under the stated institutional design, public leadership gains aggregate effectiveness when accompanied by shared objectives and coordination arrangements that account for cross-actor consequences. Residual heat-risk pressure and social loss remain normalized comparative outputs, and their event-specific health or economic interpretation requires empirical calibration.

### 6.2 Early warning as temporal governance capacity

Warning lead time determines when event-specific information becomes available for revising adaptation plans. The realized heat forcing *H*(*t*) remains fixed across values of (*L*), allowing changes in the outcomes to be attributed to the timing of the information update. Social loss, peak residual pressure, and peak effort decline as the warning arrives earlier. The largest incremental improvement in the S3 experiment occurs between (*L* = 0) and (*L* = 1), when the first pre-peak action window becomes available. Further extensions produce smaller gains because a growing share of feasible adjustment has already been undertaken. This pattern identifies usable preparation time as the central mechanism through which warning lead time affects the modeled response (6, 31).

The effort trajectories show how this mechanism operates. Warning close to the heat peak concentrates public communication, medical preparation, venue adjustment, cooling provision, and team-level measures within a short interval. Earlier information allows resources to be positioned progressively and gives adaptive capacity more time to accumulate before the strongest forcing occurs. Peak effort consequently falls and the residual-pressure trajectory develops from a better-prepared state. The Adaptation Timing Index adds a temporal qualification: its small increase from (*L* = 0) to (*L* = 1) reflects an intensified near-peak response, while longer lead times move the center of effort progressively earlier. Information receipt and temporal reorganization therefore occur at different stages of the warning response (38).

Operationally useful lead time depends on forecasting and institutional readiness. Shared triggers, assigned authority, available finance, pre-positioned resources, and rehearsed procedures determine how much of the forecast horizon remains available for action. Verification, communication, authorization, and mobilization requirements create a difference between nominal and usable lead time. The values of (*L*) in this study refer to normalized event time. Translation into hours or days requires information on the competition schedule, forecast-update process, institutional response time, and the implementation period required for each protective measure.

### 6.3 Information–capability interaction and the preparation-window boundary

The joint response surface shows that warning time and organizer capability shape the value of coordination together. A higher γ_*o*_ increases the immediate reduction in residual pressure produced by organizer effort. Nash behavior values this contribution through the organizer's objective, whereas cooperation incorporates its effects across all three objectives. The difference in marginal valuation increases the cooperative gain as organizer capability improves. Relative gain rises rapidly once a usable preparation interval becomes available and reaches its highest region around (*L* = 2–3). At longer lead times, Nash actors also make greater use of advance information, narrowing the percentage difference even as absolute social loss and peak residual pressure continue to decline. The response surface therefore separates the absolute benefit of earlier information from the additional benefit associated with coordination.

The preparation-window analysis examines how this temporal mechanism changes with the heat-shock profile. Within the tested range, $L_{80}^{†}$ responds more strongly to duration than to intensity. A higher peak amplitude increases both modeled loss and the total benefit available from warning, leaving the proportion captured at a given (*L*) comparatively stable. Greater duration causes elevated forcing to begin earlier and persist longer, shifting the benefit-capture point toward an earlier position in the event horizon. Epidemiological research also supports treating heat intensity and duration as distinct exposure dimensions (39, 40). Within the normalized planning horizon (*T* = 10), the estimated values of $L_{80}^{†}$ (2.2–2.6) denote the earliest interpolated lead times at which the reduction in cooperative social loss from its (*L* = 0) level reaches 80% of the total reduction observed between (*L* = 0) and (*L* = 6). These values therefore serve as comparative preparation-window indicators for the modeled scenarios. Translation into hours or days requires calibration to the event schedule, forecast process, local exposure conditions, population vulnerability, and intervention mobilization times.

### 6.4 Cooperation changes the composition of responsibility

Cooperation changes the composition of adaptation effort. In the baseline experiment, the organizer's share rises from 25.1% under Nash to 33.3% under cooperation, while the team's share falls from 42.3% to 36.1%. This reallocation reflects the organizer's comparatively strong direct-mitigation channel, with (γ_*o*_ = 0.20) compared with (γ_*g*_ = 0.08) and (γ_*a*_ = 0.05). Organizer effort under Nash is selected according to *J*_*o*_; the cooperative solution values the same intervention through its contribution to all three objectives. The resulting allocation places greater weight on an event-level control that produces benefits across institutional boundaries.

This distinction corresponds to the distribution of operational authority at major sporting events. Organizers can alter competition schedules, venue operations, cooling and hydration provision, crowd movement, and on-site medical arrangements. Teams have greater control over acclimation, individualized hydration, physiological monitoring, workload management, and recovery. Evidence from sport-heat research supports the contribution of both operational and athlete-level measures (7, 20, 41). The organizer's 33.3% share remains a simulated response to the specified coefficients and carries no universal allocation target. Empirical responsibility shares would depend on intervention effectiveness, implementation costs, population coverage, and institutional capacity. The difference between assigned responsibility and actor-specific gain motivates the cost-sharing analysis.

### 6.5 Cost sharing reconciles responsibility and private benefit

All three actors have lower objective values under cooperation than under Nash in the baseline case, so the cooperative allocation satisfies the participation condition before transfers. The gains are distributed unevenly. Relative to Nash, the government and team objective values fall by 2.686 and 2.939 normalized loss units, respectively, while the organizer's value falls by 0.165. The organizer therefore assumes a larger share of adaptation effort while receiving the smallest direct modeled gain. The contract addresses this distributional issue through two components: θ_*i*_*J*_Σ_ aligns each actor's marginal decision with the cooperative objective, and *K*_*i*_ allocates the cooperative surplus without changing that marginal condition (42, 43).

A practical counterpart would be an agreement established during pre-event planning. The Nash benchmark represents the status quo allocation of responsibilities, and the cooperative plan specifies the additional measures required from each institution. The resulting settlement may be implemented through a pooled heat-health contingency fund, earmarked host-authority support, reimbursement of verified venue or schedule-adjustment costs, shared medical-preparedness budgets, joint procurement, or in-kind commitments of personnel, facilities, equipment, and transport. In the baseline experiment, compensation to the organizer corresponds to the additional event-level measures assigned under cooperation, while government and team contributions reflect their allocated shares of the cooperative surplus. Comparable financing arrangements are used when implementation costs and health benefits extend across institutional boundaries (33, 44).

Implementation requires agreed heat-alert triggers, eligible activities, decision authority, expenditure records, monitoring procedures, and an enforceable settlement rule. Negotiated shares may reflect statutory responsibilities, fiscal capacity, verified costs, intervention effectiveness, affected populations, and bargaining arrangements. The equal specification (θ_*i*_ = ω_*i*_ = 1/3) provides a transparent analytical benchmark. Transfer values identify each actor's net contribution or compensation position in normalized loss units. Event-specific financial commitments would require empirical costing and agreement among the participating institutions.

### 6.6 Limitations and empirical extensions

The first limitation concerns empirical scale. Heat pressure, adaptation effort, and actor-specific losses are expressed in normalized units rather than calibrated to WBGT, UTCI, core temperature, medical encounters, service disruption, or monetary cost. The reported percentages therefore describe comparative changes within the model. Translating them into expected reductions in heat illness or financial loss would require event-level data linking environmental exposure, implemented measures, health outcomes, and institutional expenditure over time. Such calibration should also distinguish among athletes, officials, workers, volunteers, and spectators, since their exposure patterns and physiological vulnerability differ. A useful empirical design would combine time-stamped meteorological observations with medical records, operational logs, attendance data, and the actual costs of protective measures.

The three strategic actors also represent broad institutional categories. Government may encompass public health agencies, emergency medical services, municipal authorities, transport providers, and venue regulators. The organizer may include an international federation, a local organizing committee, stadium operators, broadcasters, and commercial partners. Teams differ in financial resources, medical expertise, roster characteristics, and access to acclimation facilities. Aggregation keeps the strategic structure tractable but conceals disagreements within each actor category and differences in their capacity to implement protective measures. It also places spectators, workers, officials, and volunteers inside the shared residual-risk terms rather than modeling their exposure and behavior separately. A disaggregated extension could introduce population-specific loss functions, vulnerability weights, and protection constraints, together with principal–agent relationships between governing bodies and the organizations responsible for local implementation. This would allow the distributional consequences of event heat policy to be assessed alongside aggregate welfare.

The Stackelberg analysis represents one exogenously specified hierarchy in which the government commits first. This choice captures the public authority's capacity to establish event-wide health requirements, coordinate public resources, and activate cross-agency emergency arrangements before downstream operational plans are finalized. Alternative leader assignments are substantively distinct because intervention efficiencies, effort costs, risk weights, and controlled measures vary across actors. The organizer controls scheduling and venue operations and has the highest baseline direct-mitigation efficiency, while event-specific heat information becomes available to all actors simultaneously. An organizer-led hierarchy could therefore produce a different timing and distribution of effort and a different aggregate-loss outcome. Future research should compare government-led and organizer-led games under the same heat-shock path, information structure, and admissible controls. The present Nash–Stackelberg comparison consequently applies to the government-first hierarchy and the tested parameterization.

The information experiment offers a similarly controlled representation of early warning. Event-specific heat information arrives at a single time and then becomes fully known. Operational forecasts are probabilistic, revised as the event approaches, and subject to both missed events and false alarms. Their public-health value also depends on communication, institutional uptake, and the willingness of decision-makers to act on uncertain information. Guidance on heat–health warning systems treats forecast performance, warning timeliness, stakeholder acceptance, and the connection between alerts and predefined responses as related parts of the same system (45). A stochastic extension could represent forecast distributions and repeated Bayesian updating, while a receding-horizon formulation could capture successive revisions to operational plans. These approaches would permit separate estimation of the value of warning lead time, forecast reliability, and institutional responsiveness.

A further simplification arises from representing adaptation as continuous aggregate effort. Major sporting events rely on a mixture of continuous preparation and discrete operational decisions, including mandatory cooling breaks, schedule changes, postponement, relocation, and cancellation. The thresholds governing these actions vary by sport, population, environmental index, and available medical capacity (15, 46). Implementation also involves lead times of its own: medical staff must be deployed, venue infrastructure prepared, revised schedules communicated, and contractual approval obtained. A hybrid-control model could combine continuous investments in preparedness with discrete competition decisions and explicit implementation delays. Empirical estimation would then require records of when a decision was authorized, communicated, and carried out, rather than relying solely on the formal time at which a warning was issued.

Finally, the cost-sharing results and preparation-window boundary remain tied to the numerical design. Equal values of θ_*i*_ and ω_*i*_ provide a transparent benchmark, while other bargaining rules would redistribute the cooperative surplus differently. Future research could estimate feasible sharing arrangements from host agreements, event contracts, insurance provisions, public-health budgets, and stakeholder preferences. The numerical level of $L_{80}^{†}$ is tied to the comparison between (*L* = 0) and (*L* = 6), the normalized horizon (*T* = 10), and the selected benefit-capture proportion. A 70% or 90% criterion, a different planning horizon, or alternative comparison endpoints produces a different value. Operational application would require normalized time to be mapped onto sport-specific decision calendars and combined with the mobilization time of each intervention. Validation across sports, climates, venues, and exposed population groups would then indicate whether the observed sensitivity to heat-shock duration extends beyond the present parameterization.

## 7 Conclusion

This study examined how governance structure, warning timing, adaptive capacity, and cost sharing shape extreme-heat preparedness at mega-sporting events. Across the tested normalized scenarios, cooperative governance produced the lowest aggregate modeled loss and residual heat-risk pressure because the protective effects of adaptation were evaluated across government, organizer, and team objectives. In the modeled government-first sequence, government-led Stackelberg governance produced marginally higher aggregate loss than Nash under the baseline setting and the tested one-way parameter ranges. This comparison is specific to the leader assignment, actor-specific objectives, and parameterization used in the analysis. For event governance, decision authority should be supported by shared evaluation of cross-institutional heat-health consequences.

Earlier event-specific information enlarged the usable preparation window, allowing protective effort to be staged and adaptive capacity to accumulate before the strongest heat forcing. The greatest incremental benefit arose when the first usable pre-peak interval became available, while subsequent extensions yielded smaller gains. Warning value also depended on institutional capability and the duration of elevated heat pressure. Mass-gathering preparedness plans should therefore connect forecast information with predefined triggers, assigned responsibilities, operational and medical response protocols, and resources that can be mobilized within the available time.

Cooperation also changed the distribution of adaptation responsibility by directing more effort toward organizer-controlled measures with event-wide reach, while the resulting benefits accrued across institutions. An ex-ante cost-sharing agreement can align this allocation with available resources through pooled contingency funds, reimbursement arrangements, shared medical-preparedness budgets, or in-kind contributions. Practical implementation requires agreement on eligible actions, decision authority, monitoring procedures, and settlement rules.

These findings describe comparative properties of a dimensionless, normalized model. Residual heat-risk pressure and social loss are model-based indices, and the preparation-window threshold is expressed in normalized time. Translation into heat-related illness, temperature or WBGT, monetary amounts, or operational warning periods requires event-specific data on exposure, health outcomes, schedules, intervention lead times, institutional costs, and resource constraints.

## Data Availability

The raw data supporting the conclusions of this article will be made available by the authors, without undue reservation.
